# Supplementary material for: Enabling interpretable machine learning for biological data with reliability scores
Source: PLoS Comput Biol. 2023 May 26;19(5):e1011175. doi: 10.1371/journal.pcbi.1011175 (PMC10249903; doi:10.1371/journal.pcbi.1011175)
Supplement: S1 Table — Classifiers were trained with two out of three classes and tested on all three classes. The missing class is indicated in the first column. Highlighted cells indicate comparisons where we do not expect significant differences between classes because they are both present in the training data. (PDF) [file pcbi.1011175.s001.pdf]

Table S1. Mann-Whitney test scores comparing SRS distributions for wheat classes under different models. Classifiers were trained with two out of three classes and tested on all three classes. The missing class is indicated in the first column. Highlighted cells indicate comparisons where we do not expect significant differences between classes because they are both present in the training data.

	w1 vs. w2	w1 vs. w3	w2 vs. w3
**w1 missing**	1.410136e-10**	4.924914e-09**	0.1297392
**w2 missing**	1.192306e-12**	0.2087325	1.882407e-11**
**w3 missing**	0.1335307	9.643178e-09**	1.498371e-09**
**none missing**	0.09014262	0.2087325	0.05774646

** p-value < 0.01
