# Supplementary material for: Enabling interpretable machine learning for biological data with reliability scores
Source: PLoS Comput Biol. 2023 May 26;19(5):e1011175. doi: 10.1371/journal.pcbi.1011175 (PMC10249903; doi:10.1371/journal.pcbi.1011175)
Supplement: S2 Table — (PDF) [file pcbi.1011175.s002.pdf]

**Table S2. Average SRS values and p-values for UKB cohort comparisons**

| Cohort | Mean SRS* | p-value † |
|---|---|---|
| Male Elevated | -10.04 | 1.53e-4 |
| Female Elevated | -11.14 | |
| Male Normal | -8.57 | 5.11e-34 |
| Female Normal | -10.47 | |
| European Elevated | -10.47 | 6.04e-6 |
| African Elevated | -11.90 | |
| European Normal | -9.22 | 1.50e-28 |
| African Normal | -11.10 | |

*SRS truncated to second decimal place

† calculated using a t-test: scipy.stats.ttest_ind(cohort_a_SRS, cohort_b_SRS)
