# Supplementary material for: Enabling interpretable machine learning for biological data with reliability scores
Source: PLoS Comput Biol. 2023 May 26;19(5):e1011175. doi: 10.1371/journal.pcbi.1011175 (PMC10249903; doi:10.1371/journal.pcbi.1011175)
Supplement: S3 Table — (PDF) [file pcbi.1011175.s003.pdf]

Table S3. Gaussian distributions by class and model for Figure 1C

	Model 1	Model 2	Model 3
Class 1	Mean = -1,-1<br>Covariance Array = np.array([[0.1, 0.085], [0.085, 0.1]])	Mean = -0.5,0<br>Covariance Array = np.array([[0.25,0.],[0.,0.25]])	Mean = 0.25, 0.25<br>Covariance Array = np.array([[-0.25, 0.2], [0.2, -0.25]])
Class 2	Mean = 1,1<br>Covariance Array = np.array([[0.1, 0.085], [0.085, 0.1]])	Mean = 0.5,0<br>Covariance Array = np.array([[0.25,0.],[0.,0.25]])	Mean = -0.5, -0.5<br>Covariance Array = np.array([[0.3,0.],[0.,0.3]])
