# Supplementary material for: Enabling interpretable machine learning for biological data with reliability scores
Source: PLoS Comput Biol. 2023 May 26;19(5):e1011175. doi: 10.1371/journal.pcbi.1011175 (PMC10249903; doi:10.1371/journal.pcbi.1011175)
Supplement: S4 Table — (PDF) [file pcbi.1011175.s004.pdf]

Table S4. Sample sizes for wheat dataset

	Training	Testing
wheat 1	35	35
wheat 2	35	35
wheat 3	35	35
