# Supplementary material for: Enabling interpretable machine learning for biological data with reliability scores
Source: PLoS Comput Biol. 2023 May 26;19(5):e1011175. doi: 10.1371/journal.pcbi.1011175 (PMC10249903; doi:10.1371/journal.pcbi.1011175)
Supplement: S5 Table — (PDF) [file pcbi.1011175.s005.pdf]

Table S5. Sample sizes for UKB cohorts

Cohort	Training	Testing
**Male Elevated**	**800**	**800**
**Female Elevated**	**NA**	**800**
**Male Normal**	**800**	**800**
**Female Normal**	**NA**	**800**
**European Elevated**	**800**	**800**
**African Elevated**	**NA**	**268**
**European Normal**	**800**	**800**
**African Normal**	**NA**	**800**
