# Supplementary material for: Enabling interpretable machine learning for biological data with reliability scores
Source: PLoS Comput Biol. 2023 May 26;19(5):e1011175. doi: 10.1371/journal.pcbi.1011175 (PMC10249903; doi:10.1371/journal.pcbi.1011175)
Supplement: S1 Fig — Scatterplots of each pair of attributes. Histograms showing the distribution for each individual attribute. (PDF) [file pcbi.1011175.s006.pdf]

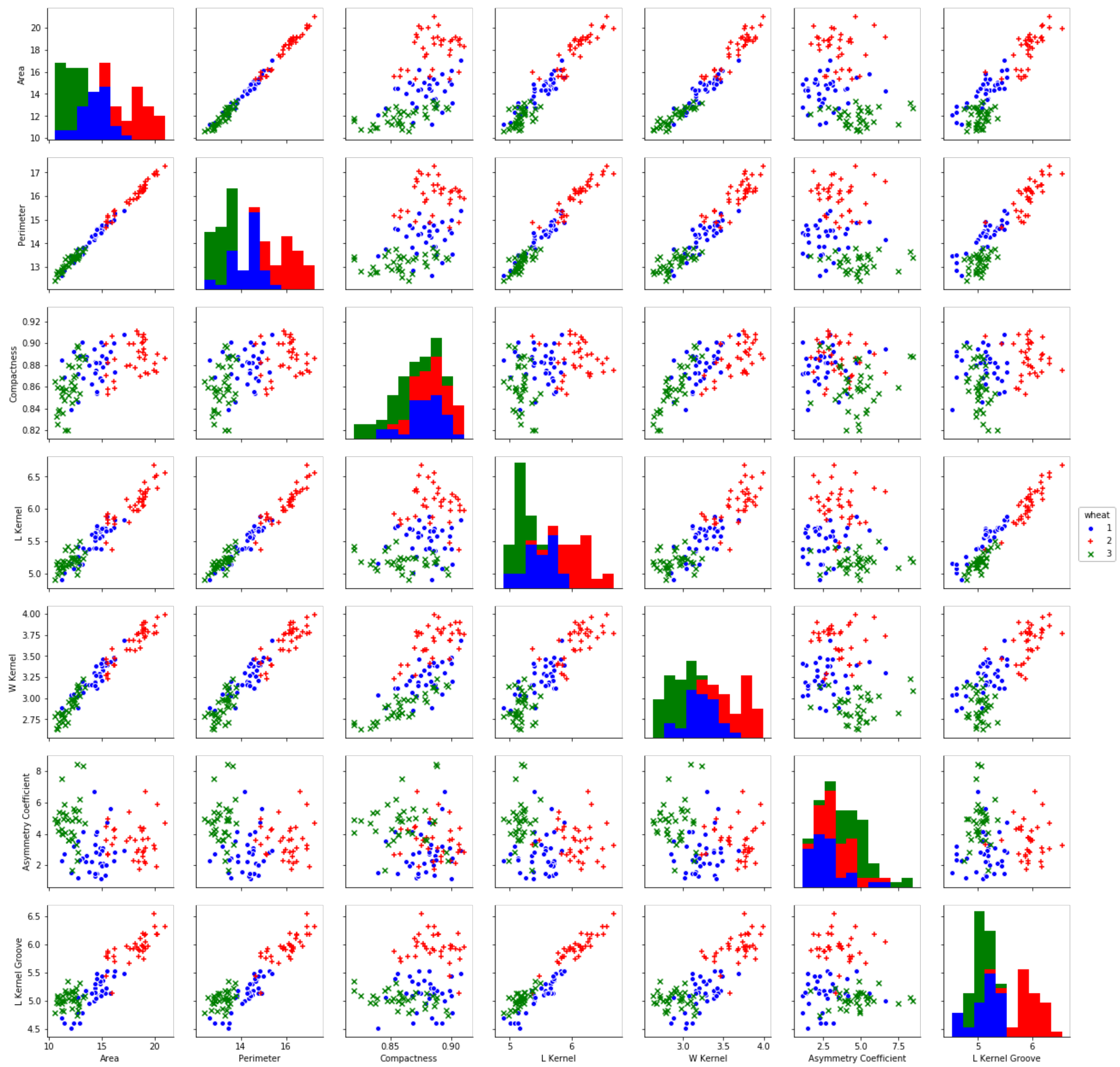

Figure S1. View of wheat dataset. Scatterplots of each pair of attributes. Histograms showing the distribution for each individual attribute.
