# Supplementary material for: Enabling interpretable machine learning for biological data with reliability scores
Source: PLoS Comput Biol. 2023 May 26;19(5):e1011175. doi: 10.1371/journal.pcbi.1011175 (PMC10249903; doi:10.1371/journal.pcbi.1011175)
Supplement: S2 Fig — Histogram of SRS scores generated when SWIF(r) was trained on all three classes of wheat, and tested on all three classes. (PDF) [file pcbi.1011175.s007.pdf]

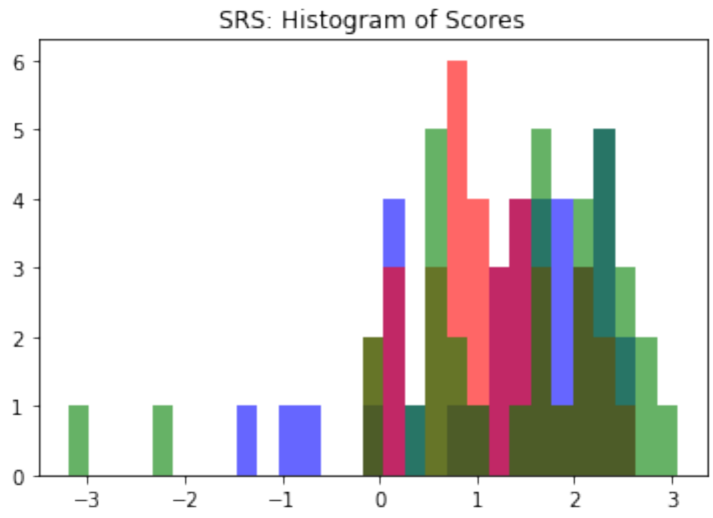

Figure S2. SRS scores show a unimodal pattern when trained on all three classes of wheat. Histogram of SRS scores generated when SWIF(r) was trained on all three classes of wheat, and tested on all three classes.
