# Supplementary material for: Enabling interpretable machine learning for biological data with reliability scores
Source: PLoS Comput Biol. 2023 May 26;19(5):e1011175. doi: 10.1371/journal.pcbi.1011175 (PMC10249903; doi:10.1371/journal.pcbi.1011175)
Supplement: S3 Fig — (Top) Receiver Operating Characteristic curve of outlier detection methods and the SRS. The “True Positive Rate” is the fraction of members of the trained classes encompassed by the score threshold, while the “False Positive Rate” is the fraction of the members of the “unknown” or excluded class encompassed by the score threshold. (Middle) The unknown class is shown in blue (wheat 1), while the classes included in training data are shown in red and green (wheat 2, wheat 3). We see that for all methods, the majority of instances of wheat 1 (blue) are to the left, receiving lower scores than instances from known classes. (Bottom) Receiver Operating Characteristic curve of Gaussian Mixture Models (GMM) outlier detection methods and the SRS. The “True Positive Rate” is the fraction of members of the trained classes encompassed by the score threshold, while the “False Positive Rate” is the fraction of the members of the “unknown” or excluded class encompassed by the score threshold. GMM10 refers to a Gaussian Mixture Model trained with 10 components, GMM5 is trained with 5 components and GMM20 is trained with 20 components. (PDF) [file pcbi.1011175.s008.pdf]

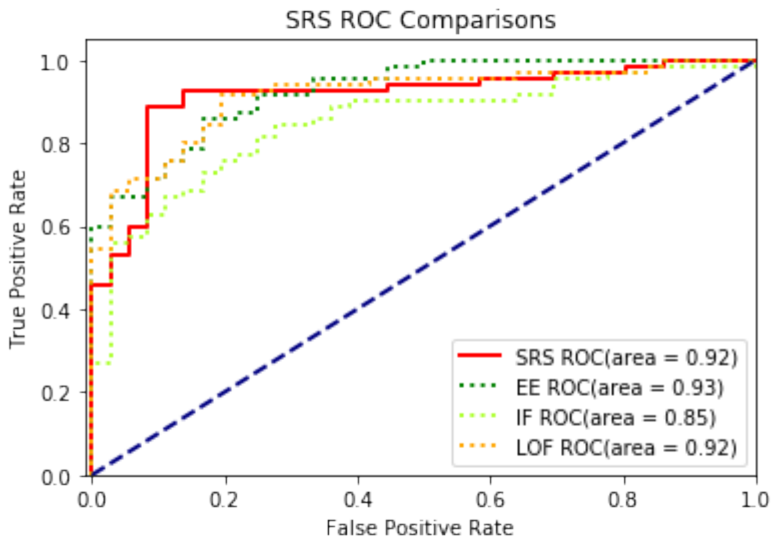
SRS ROC Comparisons
True Positive Rate
False Positive Rate
SRS ROC(area = 0.92)
EE ROC(area = 0.93)
IF ROC(area = 0.85)
LOF ROC(area = 0.92)

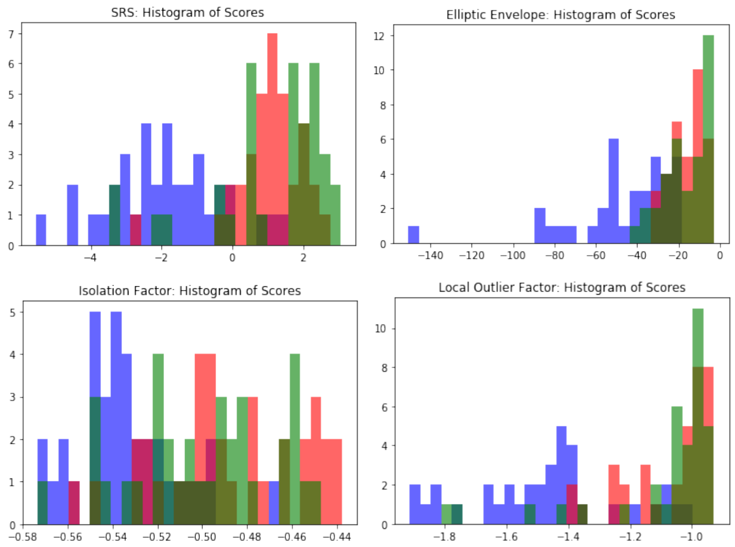
SRS: Histogram of Scores
Elliptic Envelope: Histogram of Scores
Isolation Factor: Histogram of Scores
Local Outlier Factor: Histogram of Scores

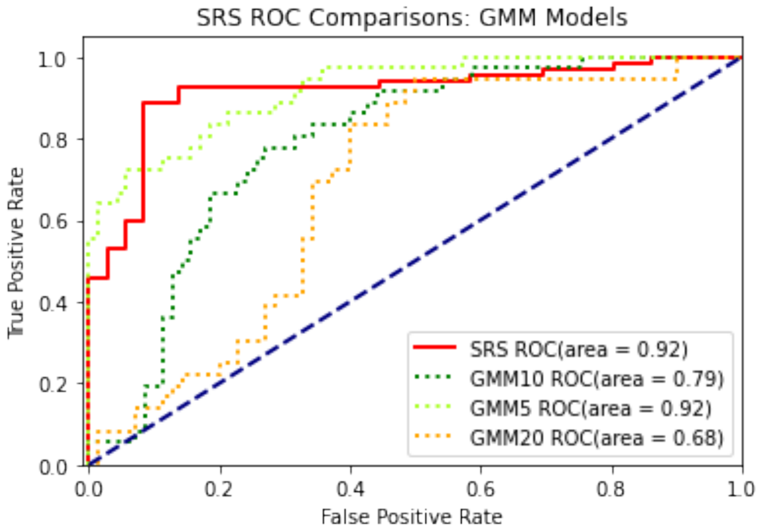
SRS ROC Comparisons: GMM Models
True Positive Rate
False Positive Rate
SRS ROC(area = 0.92)
GMM10 ROC(area = 0.79)
GMM5 ROC(area = 0.92)
GMM20 ROC(area = 0.68)

Figure S3. SRS has similar performance to other outlier detection methods when separating instances from an unknown class (wheat 1). (Top) Receiver Operating Characteristic curve of outlier detection methods and the SRS. The “True Positive Rate” is the fraction of members of the trained classes encompassed by the score threshold, while the “False Positive Rate” is the fraction of the members of the “unknown” or excluded class encompassed by the score threshold. (Middle) The unknown class is shown in blue (wheat 1), while the classes included in training data are shown in red and green (wheat 2, wheat 3). We see that for all methods, the majority of instances of wheat 1 (blue) are to the left, receiving lower scores than instances from known classes. (Bottom) Receiver Operating Characteristic curve of Gaussian Mixture Models (GMM) outlier detection methods and the SRS. The “True Positive Rate” is the fraction of members of the trained classes encompassed by the score threshold, while the “False Positive Rate” is the fraction of the members of the “unknown” or excluded class encompassed by the score threshold. GMM10 refers to a Gaussian Mixture Model trained with 10 components, GMM5 is trained with 5 components and GMM20 is trained with 20 components.
