# Supplementary material for: Enabling interpretable machine learning for biological data with reliability scores
Source: PLoS Comput Biol. 2023 May 26;19(5):e1011175. doi: 10.1371/journal.pcbi.1011175 (PMC10249903; doi:10.1371/journal.pcbi.1011175)
Supplement: S6 Fig — For each class, 800 individuals meeting the class definition (sex and HBA1C status) were selected at random from the set of individuals who self identified. PCs 1–10 are shown. (PDF) [file pcbi.1011175.s011.pdf]

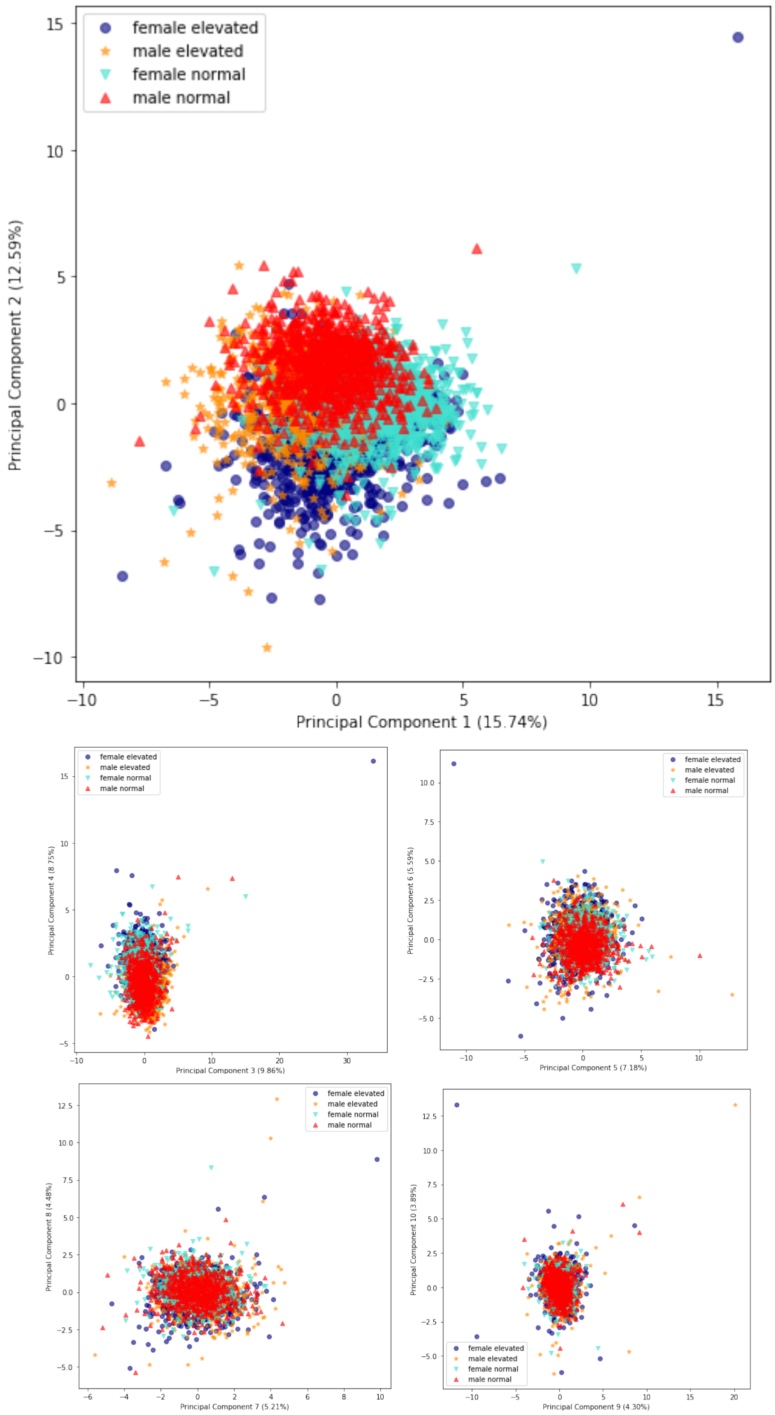

Figure S6. Principle Components Analysis of dataset composed of health attributes for male and female individuals of European descent with both normal and elevated HBA1C. For each class, 800 individuals meeting the class definition (sex and HBA1C status) were selected at random from the set of individuals who self identified . PCs 1-10 are shown.
