# Supplementary material for: Enabling interpretable machine learning for biological data with reliability scores
Source: PLoS Comput Biol. 2023 May 26;19(5):e1011175. doi: 10.1371/journal.pcbi.1011175 (PMC10249903; doi:10.1371/journal.pcbi.1011175)
Supplement: S7 Fig — For each class, 800 individuals meeting the class definition (ancestry and HBA1C status) were selected at random. Only 268 individuals met the class definition of African ancestry and elevated HBA1C, so only these 268 individuals were included for that class. PCs 1–10 are shown. Individuals of both sexes were included in the analysis. (PDF) [file pcbi.1011175.s012.pdf]

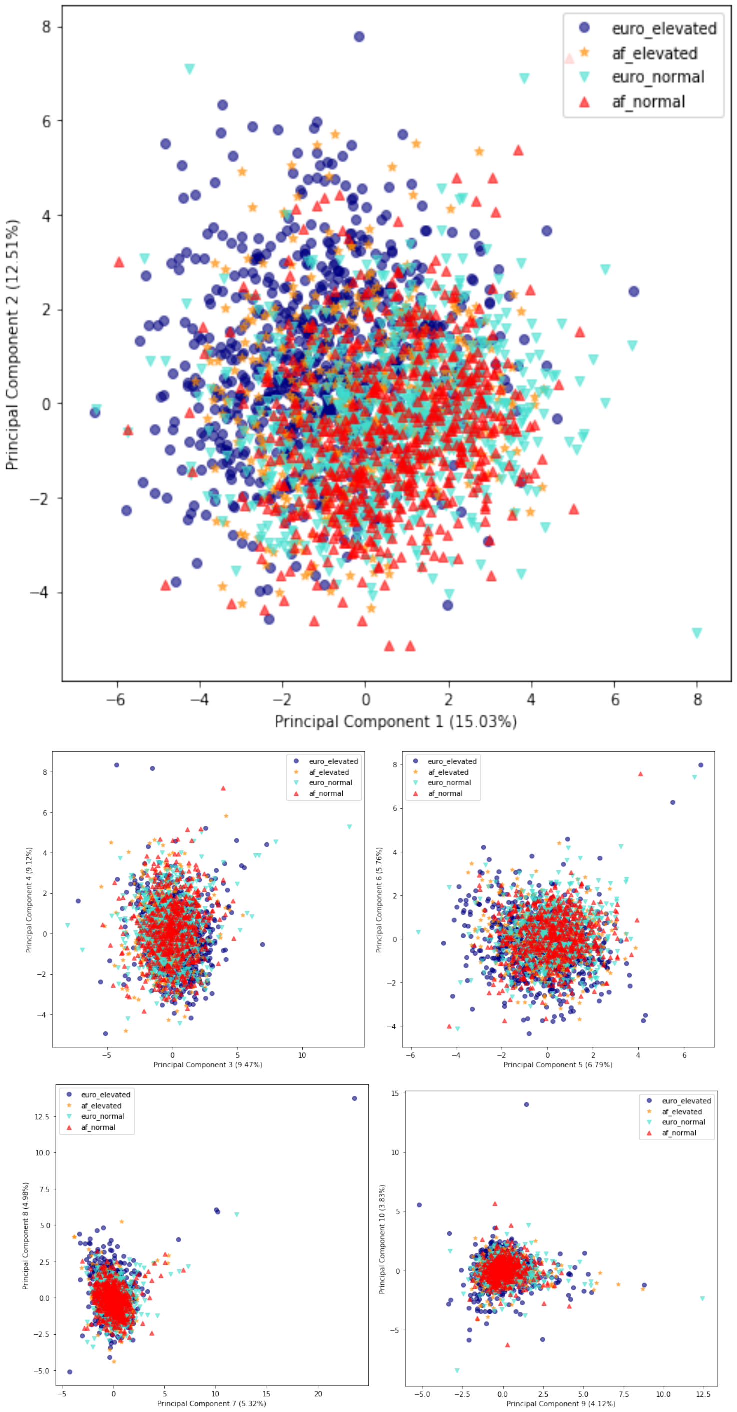

Figure S7. Principle Components Analysis of dataset composed of health attributes for individuals of both African and European descent with both normal and elevated HBA1C. For each class, 800 individuals meeting the class definition (ancestry and HBA1C status) were selected at random. Only 268 individuals met the class definition of African ancestry and elevated HBA1C, so only these 268 individuals were included for that class. PCs 1-10 are shown. Individuals of both sexes were included in the analysis.
