# Supplementary material for: Enabling interpretable machine learning for biological data with reliability scores
Source: PLoS Comput Biol. 2023 May 26;19(5):e1011175. doi: 10.1371/journal.pcbi.1011175 (PMC10249903; doi:10.1371/journal.pcbi.1011175)
Supplement: S9 Fig — Boxplots (left) and histograms (right) represent the same data, with histograms zoomed out to show outliers, while outliers are not shown in boxplots. Annotation “ns” indicates “not significant,” “*” indicates a significant difference (t-test: top: p = 0.165, bottom p = 0.00216*) (PDF) [file pcbi.1011175.s014.pdf]

Elevated HBA1C: European (purple) vs. African (pink)

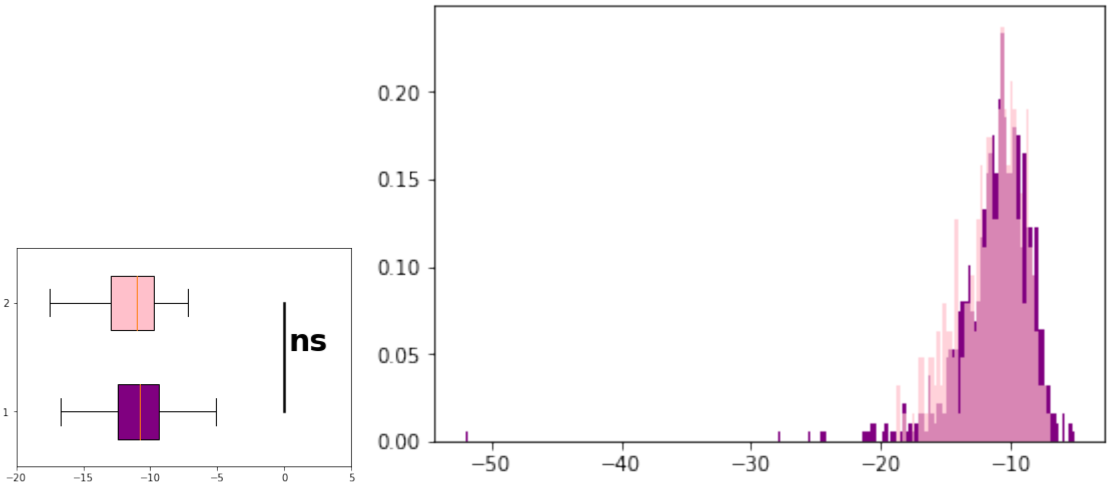

Normal HBA1C: European (purple) vs. African (pink)

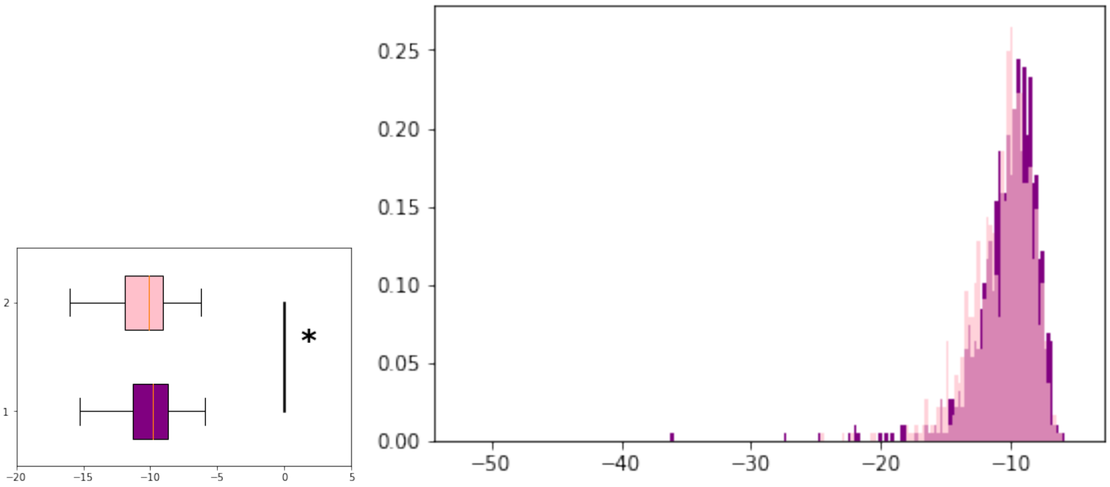

Figure S9. SWIF(r) model trained with both African and European individuals and tested on both African and European individuals. Boxplots (left) and histograms (right) represent the same data, with histograms zoomed out to show outliers, while outliers are not shown in boxplots. Annotation “ns” indicates “not significant,” “*” indicates a significant difference (t-test: top: p= 0.165, bottom p = 0.00216*)
