# Supplementary material for: Enabling interpretable machine learning for biological data with reliability scores
Source: PLoS Comput Biol. 2023 May 26;19(5):e1011175. doi: 10.1371/journal.pcbi.1011175 (PMC10249903; doi:10.1371/journal.pcbi.1011175)
Supplement: S10 Fig — A SWIF(r) model was trained with combined African and European data, and tested on the African and European test cohorts from Fig 3. Numbers correspond to the following: 0 = Elevated HBA1C, 1 = Normal HBA1C. Actual class is on the y-axis, predicted class is on the x-axis. (PDF) [file pcbi.1011175.s015.pdf]

Confusion matrix on European test set

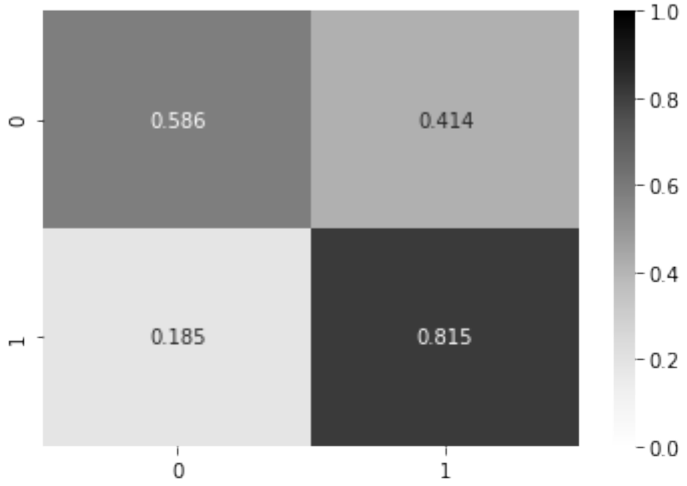

Confusion matrix on African test set

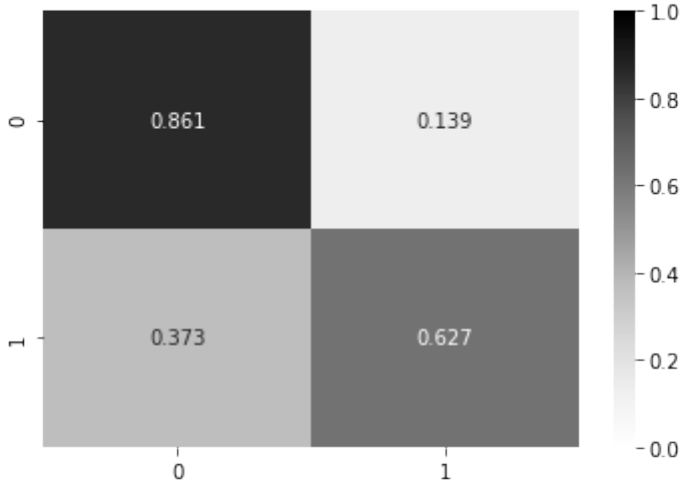

Figure S10. Confusion matrix for African and European test sets with SWIF(r) model trained with both African and European samples. A SWIF(r) model was trained with combined African and European data, and tested on the African and European test cohorts from Figure 3. Numbers correspond to the following: 0=Elevated HBA1C, 1= Normal HBA1C. Actual class is on the y-axis, predicted class is on the x-axis.
