# Supplementary material for: Enabling interpretable machine learning for biological data with reliability scores
Source: PLoS Comput Biol. 2023 May 26;19(5):e1011175. doi: 10.1371/journal.pcbi.1011175 (PMC10249903; doi:10.1371/journal.pcbi.1011175)
Supplement: S11 Fig — A SWIF(r) model was trained with combined Male and Female European data, and tested on the Male and Female European test cohorts from Fig 3. Numbers correspond to the following: 0 = Elevated HBA1C, 1 = Normal HBA1C. Actual class is on the y-axis, predicted class is on the x-axis. (PDF) [file pcbi.1011175.s016.pdf]

Confusion matrix on Male test set

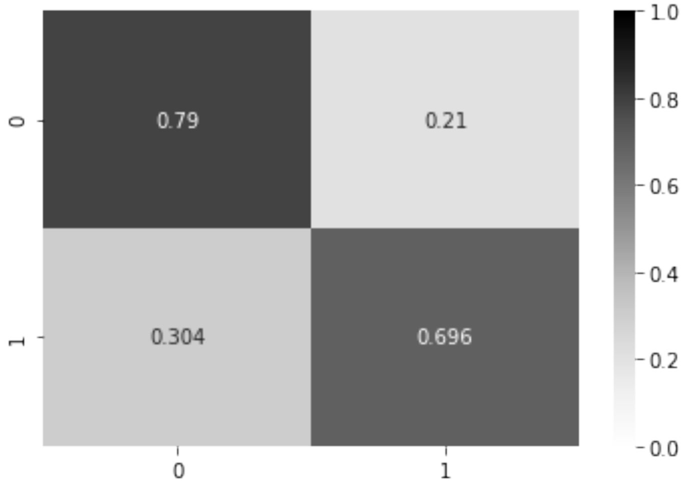

Confusion matrix on Female test set

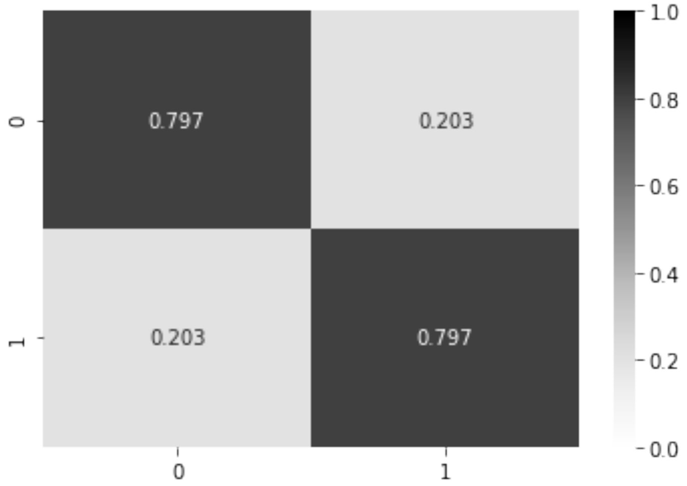

Figure S11. Confusion matrix for Male and Female European test sets with SWIF(r) model trained with both Male and Female European samples. A SWIF(r) model was trained with combined Male and Female European data, and tested on the Male and Female European test cohorts from Figure 3. Numbers correspond to the following: 0=Elevated HBA1C, 1= Normal HBA1C. Actual class is on the y-axis, predicted class is on the x-axis.
