# Supplementary material for: Enabling interpretable machine learning for biological data with reliability scores
Source: PLoS Comput Biol. 2023 May 26;19(5):e1011175. doi: 10.1371/journal.pcbi.1011175 (PMC10249903; doi:10.1371/journal.pcbi.1011175)
Supplement: Attachment — Submitted filename: Response_to_reviewers.pdf [file pcbi.1011175.s018.pdf]

Dear Dr. Sugden,

Thank you very much for submitting your manuscript "Enabling interpretable machine learning for biological data with reliability scores" for consideration at PLOS Computational Biology.

As with all papers reviewed by the journal, your manuscript was reviewed by members of the editorial board and by several independent reviewers. In light of the reviews (below this email), we would like to invite the resubmission of a significantly-revised version that takes into account the reviewers' comments.

The reviewers generally agree that the revision has improved the manuscript and we agree with their assessment. We also feel that, generally speaking, the manuscript properly acknowledges the limitations of the method. Thus, while we can see the merit in the concerns of reviewer #4, we nonetheless consider that, on balance, this paper makes a sufficient contribution to the literature that it will be of interest to many readers if the remaining issues with presentation can be fixed. The authors may decide to add new data and analyses to bolster their arguments, but we consider that the remaining issues can likely be addressed with textual improvements.

We cannot make any decision about publication until we have seen the revised manuscript and your response to the reviewers' comments. Your revised manuscript is also likely to be sent to reviewers for further evaluation.

When you are ready to resubmit, please upload the following:

[1] A letter containing a detailed list of your responses to the review comments and a description of the changes you have made in the manuscript. Please note while forming your response, if your article is accepted, you may have the opportunity to make the peer review history publicly available. The record will include editor decision letters (with reviews) and your responses to reviewer comments. If eligible, we will contact you to opt in or out.

[2] Two versions of the revised manuscript: one with either highlights or tracked changes denoting where the text has been changed; the other a clean version (uploaded as the manuscript file).

Important additional instructions are given below your reviewer comments.

Please prepare and submit your revised manuscript within 60 days. If you anticipate any delay, please let us know the expected resubmission date by replying to this email. Please note that revised manuscripts received after the 60-day due date may require evaluation and peer review similar to newly submitted manuscripts.

Thank you again for your submission. We hope that our editorial process has been constructive so far, and we welcome your feedback at any time. Please don't hesitate to contact us if you have any questions or comments.

Sincerely,

Luis Pedro Coelho

Academic Editor

PLOS Computational Biology

Sushmita Roy

Section Editor

PLOS Computational Biology

****

The reviewers generally agree that the revision has improved the manuscript and we agree with their assessment. We also feel that, generally speaking, the manuscript properly acknowledges the limitations of the method. Thus, while we can see the merit in the concerns of reviewer #4, we nonetheless consider that, on balance, this paper makes a sufficient contribution to the literature that it will be of interest to many readers if the remaining issues with presentation can be fixed. The authors may decide to add new data and analyses to bolster their arguments, but we consider that the remaining issues can likely be addressed with textual improvements.

We thank the editor for helping us improve this manuscript, and helping us navigate through the review process. We have endeavored to address the remaining comments from the reviewers, and we think the resulting manuscript is clearer and has greater impact as a result of these suggestions.

Reviewer's Responses to Questions

Comments to the Authors:

Please note here if the review is uploaded as an attachment.

Reviewer #1: The authors have addressed my comments. I have no further questions.

We thank the reviewer for their thoughtful feedback in the first round of reviews. We strongly believe that they have improved the manuscript.

Reviewer #2: - This work presents a novel, generalizable metric “reliability score” (or “SRS” in the context of the SWIF(r) classifier), which is applied to diagnose three common issues in machine learning analyses arising from differing contexts between training and testing data (missing classes, cohort effects, missing attributes). Tested on three distinct datasets, the results demonstrate the wide applicability of the SRS and reliability score concept in general at supporting machine learning analyses.

- The authors have painstakingly addressed my previous comments with additional control studies, statistical tests for major results, characterizations of input data, and clarifications in the text. Aside from minor additional contextualization and grammatical fixes, this considerably stronger manuscript is ready for publication in PLOS Computational Biology.

We thank the reviewer for their thoughtful reviews, and agree that the manuscript is stronger for them. We have done our best to address the remaining issues below.

- SRS and systemic bias: The PCA analysis (Fig S6-S7) seems to suggest the existence of clear systemic differences between Male vs. Female cohorts in attribute space, but such differences are not as obviously present when comparing European vs. African cohorts. This result should be used to contextualize why the SRS underperformed in the ancestry mismatch case when compared to the sex mismatch case.

Thank you for this comment. The following text has been added to the Results section to provide additional context to the ancestry mismatch results:

PCA analysis (Figures S6 and S7) provides an additional context for the challenge of classification in the ancestry mismatch model: while there is a clear systemic difference between the Male and Female cohorts in attribute space (Figure S6), there are no obvious differences between African and European cohorts (Figure S7). The lack of obvious differences between the African and European cohorts, and the fact that distribution shifts in this context cause individuals from one class to look like individuals from another class are both challenging situations where we might expect many classifiers to fail.

- Introduction and Discussion: Regarding discriminative models, could the SRS still be computed or estimated for a discriminative model that yields class probabilities (possibly by random sampling)? Since any discriminative model can be made to provide class probabilities through ensemble learning, this would significantly increase the generalizability of the SRS.

The design of the SRS is indeed specific to a generative model; although discriminative models can be made to provide class probabilities, as the reviewer points out, there is no way to distinguish solely from those class probabilities how reliable they are. This is the same issue we see with SWIF(r) probabilities (without the SRS). For example, a 50/50 probability assignment in a binary classification can represent a case in which the two classes are both a good fit, or neither is a good fit. In other words, the probabilities inherently represent relative fit, but not absolute fit. We have added the following text to emphasize this point in the Introduction:

"This allows a user to distinguish between a classification prediction that is made because the instance in question is a genuine match to one of the trained classes, and a prediction made because the classifier has simply found the "least bad" option."

The closest analog for the discriminative class of models, specific to neural networks, is the work of Thulasidasan *et al.* (2019 and 2021) in which the model is given the option to abstain, based on training during which the model learns to recognize features associated with instances that are unreliable (i.e. instances are systematically mislabeled). We have added an additional clause to the following excerpt (also in the Introduction) to increase clarity (bold is new):

"Abstention in the context of discriminative classifiers can be addressed with the addition of an "abstention class" in training data (Thulasidasan et al. 2021) or an "abstention option" included in the loss function (Thulasidasan et al. 2019). In the context of a generative classifier, we assert that the addition of an abstention class or abstention option is not necessary; we can instead use the data model itself (via the SRS) to identify instances where abstention is desirable **without requiring the model to learn features associated with unreliable or confusing instances**."

- Minor Issues

*Figure 2A, Panel 3: Y-axis label "Assymetry" -> "Asymmetry"

*Figure 4B: Legend “Noise Removed Neutral” is missing a colon

*Figure S3-S5, Panel 3: Title “Eliptical” -> “Elliptic”

*Figure S3, Subtitle: Missing period after “Figure S3”

We have fixed these errors, and thank the reviewer for bringing them to our attention.

Reviewer #3: The authors have added contextualization and comparisons to existing work as I requested in my review. This analysis shows that SRS performs comparably with other simpler methods. Overall, SRS/SWIF(r) seems like a single framework alternative to an otherwise multi-step process (discriminator + outlier detection), that may not necessarily perform better or faster than the multi-step process. I would suggest that the authors thoroughly discuss the limitations of their approach and address the following points:

- The biggest limitation is that SRS scales quadratically with number of features (n), which the authors haven't mentioned. This is because training and inference of AODE scales quadratically with n. This limits the use of SWIF(r)/SRS to problems with a moderate number of features, and this is reflected in the examples shown in the paper.

We agree with the reviewer that SWIF(r)/SRS are best-suited to applications with a moderate number of features; training does scale quadratically with the number of features (although application of the trained classifier is generally quite fast), but we would also argue that with very large feature counts, the pairwise correlation assumptions made by both SWIF(r) and the SRS may not be ideal in the first place, as they will not fully capture more high-dimensional relationships. On the other hand, if there are a large number of features with a high level of correlation, then users may want to perform dimensionality reduction on their feature space to improve performance, bringing the number of features down to something more moderate. We have added the following text to the Discussion to address this limitation:

“We note that the SRS, similar to SWIF(r) itself, is particularly suitable as an explanatory tool for applications with a small-to-moderate number of features. With a large number of features, pairwise correlations learned by the SWIF(r) framework may not be sufficient to capture the full structure of the data, and other tools for outlier detection or distribution shift might be more desirable. On the other hand, in situations in which the features are highly correlated, it might be preferable to reduce the number of features to a smaller, less correlated set using an approach such as PCA.”

- The authors mention "Rather than performing outlier detection or looking for distribution differences prior to performing classification, or as a post-hoc analysis, researchers get SRS output and SWIF(r) classification output in a single step.". This assumes that SWIF(r) is a competitive classification model for any task, which has not been demonstrated in the paper. In many cases, discriminative models like random forests and neural networks would give the best classification performance, and the gap between them and SWIF(r) can be large (the converse has not been demonstrated in the paper). So the claim that researchers can get classification and reliability scores in a single step is not that relevant if the classification performance is sub-par.

In the paper that introduced SWIF(r) (Sugden *et al.* 2018, *Nature Communications*), we demonstrate SWIF(r)'s performance to be competitive with methods like these. In that paper, we compared SWIF(r) to two readily-available machine learning methods for detecting selective sweeps: evoNet, which uses deep learning on selection statistics, and evolBoosting, which uses boosted logistic regression, and showed that for this task, SWIF(r) outperformed both. We expect that the best machine learning approach will vary based on the particular task and structure of the data, so we don't expect this to be the case for all applications, but we can say that SWIF(r) is at least competitive with this class of models. Furthermore, the ability of SWIF(r) to work in the presence of missing data without throwing out features or instances, or relying on imputation, is a substantial benefit that distinguishes SWIF(r) from these other methods. Its simplicity also allows for better transparency, making it less vulnerable to artifacts compared to black-box approaches such as deep learning. We agree that not all researchers will choose to use SWIF(r) over other classifiers, but we maintain that it is a valuable option in many cases. We have added a sentence to the Introduction to reference the comparative performance of SWIF(r) with other methods (bold is new):

"We previously introduced SWIF(r) (SWeep Inference Framework (controlling for correlation), a supervised machine learning classifier that we applied to the problem of classifying genomic sites as neutrally evolving or undergoing positive selection in human population genetic data (Sugden *et al.* 2018). **For this application, we show SWIF(r) to be competitive with other methods including deep neural networks and boosted logistic regression**."

- Assuming that outlier detection and classification are performed separately, one can use a cheaper outlier detection method such as LOF (with kd-tree for example) which performs as well as SRS (as per new analyses). In general, for datasets with moderate number of features and examples, it is not that cumbersome to run multiple methods (no timing data provided by the authors). Together, it suggests that one can gain better classification performance and comparable outlier detection using a combination of a discriminative method + LOF (or similar), which would undermine the need to use SRS.

Related to our response to the previous question, we note that SWIF(r) is a competitive classifier for at least some applications, but we do acknowledge that others may prefer a discriminative method depending on their situation and the structure of their dataset. In the case where a user decides to use SWIF(r), the SRS is particularly valuable because it shares the classifier's underlying framework, thereby contributing towards interpretability. In the case where a user decides to use a different classifier, we agree that running multiple methods of outlier detection is a good practice, and we propose that the SRS can be added to this class of models. We note, though, that the SRS has the added benefit of helping to visualize distribution shift between training data (perhaps simulated) and application/testing data. When using SWIF(r) and SRS together, such SRS shifts can furthermore be directly connected to difficulties that the SWIF(r) classifier might have on the application/testing data. We also note that a benefit of the SWIF(r)/SRS framework is that it makes the reliability estimation automatic. While many computationally sophisticated researchers may think to run outlier tests in conjunction with a classifier, we want this output to be automatic for the majority whose expertise lies elsewhere. In many areas of biology, it is common for researchers to use classifiers "out-of-the-box" and rely directly on their output; for these researchers, adding such a step to their pipeline might not even occur to them; we believe that prompting these researchers to consider model fit as a default is crucial for the appropriate application of these classifiers across biology. We have added the following text to the discussion to make this point more strongly (bold is new):

"By packaging SWIF(r) and the SRS together into a single workflow, we aim to give researchers the tools to both apply the machine learning method, and to critique and interpret the results they obtain. **In addition, by including the SRS as default output when using the SWIF(r) classifier, we hope to increase the adoption of this way of thinking, encouraging researchers across the biological spectrum who use out-of-the-box classifiers to interrogate results that might be too good to be true, or understand the ways in which their training data may be systematically biased.**"

- One of the advantages of SRS posited by the authors is its ability to handle missing data seamlessly. While this is a reasonable advantage, the authors should mention that an alternative to manage missing data is by using imputation methods (e.g. denoising autoencoders) followed by outlier detection methods/discrimination.

In some situations missing data can be managed using imputation methods such as denoising autoencoders. However, in some cases it is not appropriate to impute data. Examples when it is not appropriate to impute data include when a statistical feature is undefined, or calculation of a feature may be restricted, for example, some genetic linkage measures can not be calculated

near chromosome ends. In general, any method of imputation might be misleading if data are missing not at random (MNAR) (Stern et al. 2009, Lin and Tsai 2020). Imputation also may not be feasible with some small datasets or in datasets where a certain fraction of data is missing (Bodner 2008, Madley-Dowd et al. 2019) a situation where SWIF(r) is poised to perform well. The following text was added to the Introduction to reflect this observation.

SWIF(r) is able to perform classification even when instances contain missing attributes, an unusual feature in the machine learning space. While in some circumstances missing data can be imputed, SWIF(r) serves a particularly important role in circumstances when data cannot be reliably imputed, such as when data is missing in a meaningful pattern (aka. “missing not at random”)(Stern et al. 2009, Lin and Tsai 2020), or when sample size and proportion of missing data restrict imputation (Bodner 2008, Madley-Dowd et al. 2019).

Sterne, Jonathan A.C., Ian R. White, John B. Carlin, Michael Spratt, Patrick Royston, Michael G. Kenward, Angela M. Wood, and James R. Carpenter. 2009. "Multiple imputation for missing data in epidemiological and clinical research: potential and pitfalls." BMJ 338

Lin, W. C., Tsai, C. F. 2020. “Missing value imputation: a review and analysis of the literature (2006–2017).” Artif Intell Rev 53 1487–1509. https://doi.org/10.1007/s10462-019-09709-4

Bodner, T. E. 2008. “What Improves with Increased Missing Data Imputations?” Structural Equation Modeling: A Multidisciplinary Journal 15(4): 651-675. 10.1080/10705510802339072

Madley-Dowd, Paul, Rachael Hughes, Kate Tilling, Jon Heron. 2019. “The proportion of missing data should not be used to guide decisions on multiple imputation.” Journal of Clinical Epidemiology. 110: 63-73. https://doi.org/10.1016/j.jclinepi.2019.02.016

Minor:

- Corrected link that was not working in previous review: https://scikit-lego.netlify.app/mixture-methods.html#outlier-detection. This shows a method very similar to SRS (without class labels).

We added comparison to the above method to Figures S3-S5, and now reference it in the text in the Materials and Methods and the Results. The performance of this method was generally comparable to the SRS and other methods used for comparison. The GMM method did have superior performance with certain test data and component-number selections (see Figure S4 and S5). However, in many cases the performance was much worse than the SRS or other methods, depending on the user-provided number of components, resulting in poor performance in other circumstances (Figure S3 and S5). There was no universally best number of components to choose for performance comparison, so three variations of the model were

included in testing. The method does have a very similar mathematical basis to SWIF(r) and the SRS, and was useful to consider in this context. SWIF(r) benefits from automatic selection of component number, which simplifies user decisions when running SWIF(r) and the SRS compared to this GMM model.

- "Briefly, from simulated data for each class, SWIF(r) learns a joint, two-dimensional Gaussian mixture for each pair of.." (Page 9)-- "from simulated data" is not a requirement

We thank the reviewer for catching this. We have replaced “simulated data” with “training data.”

- I could not find information on how exactly SRS is calculated in the case of missing data. Also how will one account for cases where there are different number of missing variables in different examples?

We have added text to Materials and Methods (Overview and Definition of SRS) to explain how both SWIF(r) and the SRS are calculated in the presence of missing data. In short, both are composed of sums and products over a set of n attributes, including all single and pairwise conditional distributions; in the case that a subset of attributes are missing, each measure is simply calculated on the set of present attributes, reducing the number of objects in each sum and product. The new text is as follows:

“In the case of an instance with one or more missing attributes, both SWIF(r) and the SRS are calculated based on the set of present attributes rather than the full set of n attributes, altering the number of density functions in each sum and product of Equations 2 and 3.”

To the question about accounting for different numbers of missing attributes across different examples, it is a feature of both SWIF(r) and the SRS that this does not pose a problem. From one instance to the next, each measure can be calculated based on the available information for that particular instance. The SRS then helps to understand which instances are missing too many informative statistics for the SWIF(r) probability to be reliable. We have added text to the Results (“The SRS can be used to filter instances missing valuable attributes” section) to make this more explicit:

“SWIF(r) allows for any number of missing attributes at a given instance, as long as at least a single attribute is defined, and it allows for the number of missing attributes to vary from instance to instance.”

Reviewer #4: After reading through the authors’ response, their measure is an outlier detection approach that does not fare better than what’s already out there. In addition, while the authors have provided additional information, I do not see convincing evidence is presented that that their approach allows instances from unknown classes and systemic mismatches between training/testing set to be distinguished. My response to the authors’ replies started with “Reviewer response”.

Reviewer #4:

First, there is no question that, when we know the ground truth in either cases, SRS can help

distinguish the presence of an unknown class, or indicate the presence of systemic bias.

However, one of the most challenging issues is that frequently we cannot distinguish 1) [an unknown class present in testing data that was not present in training data], 2) [systemic mismatch between training and testing data]. When we have poorly classified test instances, we almost always do not know if it is a new class or the training data simply cannot lead to a more general model. My understanding is that SRS cannot help us in this regard. So, the claim that SRS can help resolve 1) and 2) only applies

when we know a prior what the situation is, which make it not particularly useful.

We have worked to address these comments in a few ways. First, we think there are particular instances in which the distribution of the SRS itself can provide some clues. In particular, the following is an excerpt from The SRS is sensitive to instances from unknown classes:

"We note the roughly bimodal shape of the SRS scores when a class missing from training data is represented by many instances in testing data. Combining the bimodal profile with domain expertise to inspect the low-SRS samples and identify their common features could lead to the identification of a novel class."

Reviewer response: Looking into the section where this is explained, it is unclear where the authors present the evidence supporting the above observation. In the sentence prior, Figure S2 is mentioned but it is about a unimodal pattern of SRS score.

We thank the reviewer for finding this error. The evidence is in figures S3-S5, and the text in this section now reflects this.

In addition, the question of systemic bias is often one that can be plausibly predicted; for example, in moving from simulated testing data to real-world data, or in trying to use a trained classifier on a new testing set from a new population or source. In this case, the distribution of SRS values can serve to assess the generalizability of the trained classifier. The following is an

excerpt from The SRS is sensitive to systemic bias in training data relative to application data:

“This experiment represents an important use case for the SRS; in many contexts, researchers may be unsure how well their classifiers will generalize to a new dataset. The SRS provides a quick way to view the overall difference in model fit between a training or validation set and a testing set, making it straightforward to test for significant shifts in SRS distribution using two-sample tests (Rabanser et al. 2019), and to subsequently make decisions about the need for additional training.”

Reviewer response: The above discussion seems reasonable and would have been convincing should the authors provide evidence that they can readily predict systemic biases with SRS. But this is not presented in the above response.

We illustrate this ability of SRS to detect systemic bias in the UK Biobank example. For both the sex- and ancestry-mismatch case, Figure 3A and Table 2 show that there are statistically significant shifts in SRS, with the average SRS being lower for the mismatch case than the match case. These display items are currently referenced in the text, and we have further added the following text to the end of the Results section (“The SRS is sensitive to systemic bias in training data relative to application data”) to make this more prominent (bold is new):

“The SRS provides a quick way to view the overall difference in model fit between a training or validation set and a testing set, making it straightforward to test for significant shifts in SRS distribution using two-sample tests (Rabanser et al. 2019). **In this example, we are able to observe a statistically significant decrease in SRS score on cohorts mismatched on either sex or ancestry. This analysis is crucial for making subsequent decisions** about the need for additional training.”

In situations that do not align with one of the above cases, we agree that this is a fundamentally

difficult problem, and one that really relies on domain-specific knowledge and thoughtful use of the SRS. We have added text to the Discussion that explores the nuances of this issue:

"One limitation of the SRS is that while the SRS indicates problems with missing data or deficiencies in the dataset, a solution to those problems may not always be possible or simple to implement. Researchers will need to rely on domain-specific knowledge to decide whether the indicated problem can be solved by removing instances, adding additional classes or attributes to their model, or by including more, or more diverse instances, in their training data. It may also be advantageous to train independent classifiers for cohorts that may have distinct underlying properties, for example, training separate classifiers for each sex in a study where the goal is to distinguish individuals with a disease phenotype (Mostafavi et al. 2020, Figure 3). Discovering a solution to low-reliability instances will require answers to subjective and context-dependent questions that may be difficult to anticipate and may not generalize. Care should be taken to use abstention, or the removal of instances in a principled and appropriate manner. When there is risk involved in making the wrong classification, abstention can be an important practice, as in the medical context (Herbei and Wegkamp 2009; Gandouz et al. 2021). In other cases there may be a distinctive factor impacting the performance of the classifier on specific instances: for example, when simulated training data fails to capture dynamics in unusual genomic regions. Abstention or removal of instances should not be used to boost measurement of performance of the classifier, but to invite further investigation and scrutiny. When distribution shifts or additional classes are suspected, researchers may face decisions about the number and types of classes to include in training, and cost-benefit considerations when deciding whether to gather the additional data required to add new attributes or additional training examples. Increasing transparency and publication of models with negative results would help address some of the problems raised by these subjective questions [...]

Domain-specific biological knowledge currently provides the best available guide for navigating the complex, multi-factorial choices involved in model design. In order to best leverage this domain-specific expertise, we need to ensure that machine learning models are easy to interpret, dissect and critique, even by researchers without machine learning expertise."

Reviewer response: The above detailed discussion is certainly helpful. Nonetheless, my original question remains: where is the evidence supporting SRS's capability in distinguishing 1) an unknown class present in testing data that was not present in training data, 2) systemic mismatch between training and testing data?

We have refined the manuscript in response to earlier comments from this reviewer to make two points more clear: that we can identify a missing class when it leads to a bimodal distribution of SRS scores, and that we can use two-sample tests on SRS distributions to detect a shift that may signify a systemic mismatch. We hope that these clarifications help to address this question. Of course, we agree that this is an extremely difficult problem in general, and one that deserves attention in future research. We have added the following text to the Discussion to consolidate these ideas (bold is new):

The SRS allows a user to probe for important deficiencies in the training and testing data, including missing classes, systemic mismatch between training and testing data, and instances missing informative attributes. **For example, users may look for a bimodal distribution in SRS values to indicate a large number of instances that may come from an unknown class, allowing for subsequent investigation of what those instances have in common. Additionally, analyzing SRS distributions between known subgroups may help users diagnose a systemic mismatch between training and application data.** While **a full interpretation of** the causes **contributing** to a low SRS requires domain specific knowledge, use of the SRS offers researchers several potential remedies, including removal of outliers, measurement of distribution shifts, expanding or revising the training set, or the creation of multiple classifiers to account for distinct subgroups in their analysis.

Second, the SRS appears useful in identifying instances that do not resemble training data.

However there are many other measures one can use, including very simple ones such as Euclidean distance or similarity measures like correlation, to achieve the same goal. SRS' calculation requires priors and calculation can be involved. Is it better than the other approaches? If so, can there be some ways to formally demonstrate this?

Thanks to this comment, and similar comments from the other reviewers, we now include comparisons to other methods that perform outlier or novelty detection. Please refer here to our response to point #3 from Reviewer #1, which includes references to the new display items (Figures S2, S3, and S4) in which we compare the SRS to three methods for novelty detection, as well as excerpts from the Introduction, Results, and Discussion where discuss the relationship between the SRS and outlier/novelty detection methods, methods for detection of distribution shift, and the literature about abstention in machine learning classifiers.

Reviewer response: The best practice is to provide response to each reviewer individually as frequently the motivations and suggestions are distinct which is true in this case. In addition, with the very long response document, it is a bit challenging to find what the authors are referring to. In any case, looking into the authors' response to the other reviewer, they concurred that the approach "has comparable performance". Thus, while the approach is capable of outlier detection, it does not do worse but not better either. It is a nice feature that SRS can deal with missing data. But apparently this ability does not contribute to better performance. This call into the question the significance of emphasizing SRS's ability in this regard.

We thank the reviewer for this feedback, and we agree that it is difficult to define improved performance in the presence of missing data. The other methods cannot be used at all in the presence of missing data, so any direct comparison is impossible. However, any ability to calculate a measure of fit as with the SRS is inherently valuable. To operate on such a dataset with the other methods, users need to either deliberately drop instances or features, or they need to impute the missing data, which can be difficult to do well, especially when features are missing not at random, as is often the case in real-world applications.

Third, I would love to have some reliability measure for each instance in my classification tasks. But given the first point, while I appreciate the conceptual basis of a reliability score, SRS

seems to be an outlier detection measure where there are already plenty.

We hope our answers above support the utility of the SRS. In particular, we point to a couple of excerpts from the Introduction and Discussion where we make this argument: “By packaging our classifier and the SRS together, we prioritize and highlight the need for evaluations of outliers and distribution differences to be included in every machine learning analysis. Rather than performing outlier detection or looking for distribution differences prior to performing classification, or as a post-hoc analysis, researchers get SRS output and SWIF(r) classification output in a single step. Exclusion of outliers and many out-of-distribution instances can be performed by setting a threshold on the SRS, in much the same way that other methods for outlier and novelty detection can be applied. The SRS can also be used to measure distribution shifts, fitting into the “Failing Loudly” framework for machine learning described in Rabanser et al. 2019, in which the authors advocate for machine learning tools that give warnings when facing unexpected input. In this particular framework, the SRS can serve the role of dimensionality reduction, a step in the Failing Loudly pipeline prior to testing for significant distribution shift with subsequent two-sample tests.”

“The reliability score shares some features with outlier detection, novelty detection and detecting out-of-distribution instances (Rousseeuw and Van Driessen, 1999; Breunig et al. 2000; Liu et al. 2008, Liu et al. 2012, Pimentel et al. 2014, DeVries and Taylor 2018, Lee et al. 2018), as well as methods for testing distribution shifts (Rabanser et al. 2019) [...] The SRS is able to operate in the presence of missing attribute values, a strength that sets it apart from many related methods [...] Across many areas of biology there is an opportunity to improve machine learning analyses by creating community standards and best practices for simulation, interpretability and reproducibility. By packaging SWIF(r) and the SRS together into a single workflow, we

aim to give researchers the tools to both apply the machine learning method, and to critique and interpret the results they obtain.”

Reviewer response: While a merit of the study as the authors argue is in packaging classification and outlier detection in one setting, one can also argue that such package can be unnecessary since the outlier detection task is not onerous and can be automated easily by any ML practitioner. I see the point about the need to fail loudly but I just am not sure SRS is doing this in ways that the authors have claimed.

We agree with the reviewer that ML practitioners will have an easy time running any number of outlier methods, and for those individuals, we would propose the SRS as an addition to that suite of tools. We note, though, that the SRS has the added benefit of helping to visualize distribution shift between training data (perhaps simulated) and application/testing data. Perhaps more importantly, however, we believe that an important benefit of the SWIF(r)/SRS framework is that it makes the reliability estimation automatic. While many sophisticated ML practitioners may think to run outlier tests in conjunction with a classifier, we want this output to be automatic for the majority whose expertise lies elsewhere. In many areas of biology, it is common for researchers to use classifiers “out-of-the-box” and rely directly on their output; for these researchers, adding such a step to their pipeline might not even occur to them; we believe that prompting these researchers to consider model fit as a default is crucial for the appropriate application of these classifiers across biology. We have added the following text to the discussion to make this point more strongly (bold is new):

“By packaging SWIF(r) and the SRS together into a single workflow, we aim to give researchers the tools to both apply the machine learning method, and to critique and interpret the results they obtain. **In addition, by including the SRS as default output when using the SWIF(r) classifier, we hope to increase the adoption of this way of thinking, encouraging researchers across the biological spectrum who use out-of-the-box classifiers to interrogate results that might be too good to be true, or understand the ways in which their training data may be systematically biased.**”

Have the authors made all data and (if applicable) computational code underlying the findings in their manuscript fully available?

The PLOS Data policy requires authors to make all data and code underlying the findings described in their manuscript fully available without restriction, with rare exception (please refer to the Data Availability Statement in the manuscript PDF file). The data and code should be provided as part of the manuscript or its supporting information, or deposited to a public repository. For example, in addition to summary statistics, the data points behind means, medians and variance measures should be available. If there are restrictions on publicly sharing data or code —e.g. participant privacy or use of data from a third party—those must be specified.

Reviewer #1: Yes

Reviewer #2: Yes

Reviewer #3: Yes

Reviewer #4: Yes

PLOS authors have the option to publish the peer review history of their article (what does this mean?). If published, this will include your full peer review and any attached files.

If you choose "no", your identity will remain anonymous but your review may still be made public.

Do you want your identity to be public for this peer review? For information about this choice, including consent withdrawal, please see our Privacy Policy.

Reviewer #1: No

Reviewer #2: No

Reviewer #3: No

Reviewer #4: No

Figure Files:

While revising your submission, please upload your figure files to the Preflight Analysis and Conversion Engine (PACE) digital diagnostic tool, https://pacev2.apexcovantage.com. PACE helps ensure that figures meet PLOS requirements. To use PACE, you must first register as a user. Then, login and navigate to the UPLOAD tab, where you will find detailed instructions on how to use the tool. If you encounter any issues or have any questions when using PACE, please email us at figures@plos.org.

Data Requirements:

Please note that, as a condition of publication, PLOS' data policy requires that you make available all data used to draw the conclusions outlined in your manuscript. Data must be deposited in an appropriate repository, included within the body of the manuscript, or uploaded as supporting information. This includes all numerical values that were used to generate graphs, histograms etc.. For an example in PLOS Biology see here: http://www.plosbiology.org/article/info%3Adoi%2F10.1371%2Fjournal.pbio.1001908#s5.

Reproducibility:

To enhance the reproducibility of your results, we recommend that you deposit your laboratory protocols in protocols.io, where a protocol can be assigned its own identifier (DOI) such that it can be cited independently in the future. Additionally, PLOS ONE offers an option to publish peer-reviewed clinical study protocols. Read more information on sharing protocols at https://plos.org/protocols?utm_medium=editorial-email&utm_source=authorletters&utm_campaign=protocols
