# Supplementary material for: Enabling interpretable machine learning for biological data with reliability scores
Source: PLoS Comput Biol. 2023 May 26;19(5):e1011175. doi: 10.1371/journal.pcbi.1011175 (PMC10249903; doi:10.1371/journal.pcbi.1011175)
Supplement: Attachment — Submitted filename: SRS_responses.docx [file pcbi.1011175.s017.docx]

Dear Dr. Sugden,

Thank you very much for submitting your manuscript "Enabling interpretable machine learning for biological data with reliability scores" for consideration at PLOS Computational Biology. We apologize for the delay in handling your manuscript.

As with all papers reviewed by the journal, your manuscript was reviewed by members of the editorial board and by several independent reviewers. In light of the reviews (below this email), we would like to invite the resubmission of a significantly-revised version that takes into account the reviewers' comments.

We ask that the authors address all the concerns raised by the reviewer, but note that **a common theme is a lack of comparison to existing methods and approaches**.

We cannot make any decision about publication until we have seen the revised manuscript and your response to the reviewers' comments. Your revised manuscript is also likely to be sent to reviewers for further evaluation.

When you are ready to resubmit, please upload the following:

[1] A letter containing a detailed list of your responses to the review comments and a description of the changes you have made in the manuscript. Please note while forming your response, if your article is accepted, you may have the opportunity to make the peer review history publicly available. The record will include editor decision letters (with reviews) and your responses to reviewer comments. If eligible, we will contact you to opt in or out.

[2] Two versions of the revised manuscript: one with either highlights or tracked changes denoting where the text has been changed; the other a clean version (uploaded as the manuscript file).

Important additional instructions are given below your reviewer comments.

**Please prepare and submit your revised manuscript within 60 days**. If you anticipate any delay, please let us know the expected resubmission date by replying to this email. Please note that revised manuscripts received after the 60-day due date may require evaluation and peer review similar to newly submitted manuscripts.

Thank you again for your submission. We hope that our editorial process has been constructive so far, and we welcome your feedback at any time. Please don't hesitate to contact us if you have any questions or comments.

Sincerely,

Luis Pedro Coelho

Associate Editor

PLOS Computational Biology

Sushmita Roy

Deputy Editor

PLOS Computational Biology

***********************

We ask that the authors address all the concerns raised by the reviewer, but note that a common theme is a lack of comparison to existing methods and approaches.

We thank the editor for these reviews and for the time to address them fully. Thanks to this feedback, we believe that the manuscript is much stronger. In particular, we have implemented multiple other novelty detection algorithms for the purposes of performance comparison on detecting missing classes in training, and we demonstrate how two-sample tests can be used downstream of the SRS for detection of distribution shifts. We place the SRS in the context of the broader field of abstention in machine learning classifiers, and we have implemented the controls for experiments recommended by the reviewers. Throughout the document, we have strived to increase clarity and context.

Reviewer's Responses to Questions

**Comments to the Authors:**

**Please note here if the review is uploaded as an attachment.**

Reviewer #1: Title: Enabling interpretable machine learning for biological data with reliability scores

Summary

The manuscript proposes a reliability score, SWIF(r) reliability score (SRS), to assess a Machine learning classifier’s ability to produce a reliable classification for test data. SRS builds on a previous generative model SWIF(r) from the same group which produces a posterior probability for each class in the classification task. SRS in comparison focuses only on the estimated likelihoods. The utility of SRA is shown using three common scenarios from machine learning: 1) presence of a new class in test data absent from the training data, 2) distributional mismatch between training and test data, and 3) missing attributes (features) in test data. The first scenario is tested using a classification task to distinguish between three wheat seed varieties using seed morphology features. Training data consists of two seed types and test data contains all three. SRS assigns low reliability to predictions of the wheat seed type missing from the training data whereas SWIF(r) probability confidently assigns them to the other two classes for the majority of test instances. The second scenario is showcased with UK Biobank data, where the train and test data have a demographic mismatch (training on male individuals of European ancestry for elevated versus normal sugar and testing on either sex-mismatched or ancestry mismatched data). Instances from matched data (European male) result in a higher reliability score in comparison to mismatched (sex and ancestry) instances. The final scenario is illustrated by showing that SRS can deal with missingness in the test data and instances where informative features are removed have lower SRS than instances where noise features are removed.

Major comments:

1. The code repository is not available (<https://github.com/ramachandran-lab/SRS_paper>).

The code repository is now public, and contains information for replicating the results in the paper (with the exception of the UK Biobank data, which are available under managed access at https://www.ukbiobank.ac.uk/.

2. While the SRS score is appealing to estimate the reliability of a model’s prediction, it is limited in application because of its dependence on the SWIF(r) generative model. Can it be extended to be applicable to other model types?

We thank the reviewer for the prompt to discuss this at more length, and we have added text to the **Discussion** that addresses this. Briefly, while we would argue that the generative nature of the underlying classifier is key to the design of the SRS, and is inherently a benefit with respect to interpretability, we also understand that it is sometimes desirable to use discriminative classifiers and other less interpretable approaches. We suggest a post-hoc approach in this case, either with other outlier/novelty detection methods or tests for distribution shift, or with the SRS itself, which has the benefit of being able to be calculated in the presence of missing data. See in particular the excerpt below, which contains new text expanding on this:

“The SRS represents a valuable measure for SWIF(r), and similar reliability scores could be created for closely related methods with a generative basis. This underscores the inherent interpretability of generative methods: because they learn the underlying distribution of data for each class, reliability scores can reveal elements of that process in order to improve the quality of classification, and understand its basis. Since discriminative classifiers learn decision boundaries directly without learning underlying distributions, these methods (including most neural networks, support vector machines, random forest classifiers, regression-based methods and more) require a different approach, such as post-hoc use of novelty detection methods and measures of distribution shifts. In such a case, it is possible to use the SRS independent of applying SWIF(r), as a complementary read-out alongside another machine learning classifier. In this case, the SRS would be an explanatory tool for examining results or refining the design of the training set, but could not be understood as providing insight into the specific classifier.”

3. The manuscript is lacking in any sort of comparison with existing methods for estimating uncertainty in a classifier’s prediction (For example, <https://arxiv.org/pdf/1805.11783.pdf>). Furthermore, there are several published papers about the calibrating for the distributional shift between train and test data (for example, <https://proceedings.neurips.cc/paper/2019/file/846c260d715e5b854ffad5f70a516c88-Paper.pdf>). Comparing against at least some of them will give us an idea of the utility of SRS.

We thank the reviewer for pointing us in the direction of this literature. Thanks to these suggestions and those of the other reviewers, we chose three methods that made for a straightforward comparison: Local Outlier Factor, Isolation Forest, and Elliptic Envelope. We evaluate the performance of the four methods at the task of detecting instances from a class that is not represented in training (i.e. novelty detection). We find in general that the SRS performs on par with these three methods, with the added benefits of reflecting the underlying workings of the classifier itself, and being able to work with missing data (see new display items **Figures S2, S3, and S4**.) This is referenced in the excerpt below:

**The SRS is sensitive to instances from unknown classes:**

“The “missing class” task is analogous to outlier or novelty detection, which can be evaluated by other methods including Local Outlier Factor (Breunig et al. 2000), Isolation Forest (Liu et al. 2008, Liu et al. 2012) and Elliptic Envelope (Rousseeuw and Van Driessen, 1999). We find that the SRS has comparable performance to these methods on the task of distinguishing instances that came from a class that was not included in training (Figure S3-S5) , while having the advantage of being produced automatically within the SWIF(r) pipeline with the same underlying statistical framework. One additional advantage of the SRS over alternative methods, which we explore later in this manuscript, is that it can be applied even when instances contain missing values for some attributes; none of the other methods used in this comparison allow for any missing attribute values.”

With respect to distribution shift, we argue that the SRS provides a principled way to perform dimensionality reduction prior to testing for distribution shifts in training and testing/application data. The SRS is therefore not itself a test of distribution shift; rather it enables us to perform such tests on one-dimensional distributions. Indeed, we demonstrate this by performing two-sample tests on our data including Mann-Whitney and t-tests where appropriate (see new display items **Table S1**, **Figures S8** and **S9**, and p-values throughout the manuscript). In general, we have worked to incorporate the existing research referred to here throughout the manuscript, in order to place the SRS fully in context. Please see in particular the following excerpts:

**Introduction:**

“Ideally, machine learning workflows should include steps that evaluate the fit between training and testing data: testing for outliers and distribution differences (including out-of-distribution instances and distribution shifts) between training data and testing data (Pimentel et al. 2014; Rabanser et al. 2019). This has received particular attention in the context of neural networks (Liang et al. 2017; DeVries and Taylor 2018; Lee et al. 2018), but in the context of other types of classifiers, tools available for the process of outlier detection or detecting distribution differences typically rely on different mathematical frameworks than the machine learning model and require an independent analysis. For example, one can detect outliers using the density of instances with the measure Local Outlier Factor (Breunig et al. 2000), then subsequently apply a machine learning method that relies on a neural network to make classifications. Researchers have to rely on their own knowledge and discretion to decide which, if any, evaluations to include in their experiments.

Here, we introduce the SWIF(r) reliability score (SRS) with the goal of contributing to the goals of interpretability and best-practices in machine learning workflows. We seek to simplify the decision about how to include testing for outliers and distribution differences in a few ways. By packaging our classifier and the SRS together, we prioritize and highlight the need for evaluations of outliers and distribution differences to be included in every machine learning analysis. Rather than performing outlier detection or looking for distribution differences prior to performing classification, or as a post-hoc analysis, researchers get SRS output and SWIF(r) classification output in a single step. Exclusion of outliers and many out-of-distribution instances can be performed by setting a threshold on the SRS, in much the same way that other methods for outlier and novelty detection can be applied. The SRS can also be used to measure distribution shifts, fitting into the “Failing Loudly” framework for machine learning described in Rabanser et al. 2019, in which the authors advocate for machine learning tools that give warnings when facing unexpected input. In this particular framework, the SRS can serve the role of dimensionality reduction, a step in the Failing Loudly pipeline prior to testing for significant distribution shift with subsequent two-sample tests[…]

[…]Designing abstention into classifiers should be considered a best practice (Herbei and Wegkamp 2009), especially for biomedical data (Gandouz et al. 2021). Abstention in the context of discriminative classifiers can be addressed with the addition of an “abstention class” in training data (Thulasidasan et al. 2021) or an “abstention option” included in the loss function (Thulasidasan et al. 2019). In the context of a generative classifier, we assert that the addition of an abstention class or abstention option is not necessary; we can instead use the data model itself (via the SRS) to identify instances where abstention is desirable.”

**Discussion:**

“The reliability score shares some features with outlier detection, novelty detection and detecting out-of-distribution instances (Rousseeuw and Van Driessen, 1999; Breunig et al. 2000; Liu et al. 2008, Liu et al. 2012, Pimentel et al. 2014, DeVries and Taylor 2018, Lee et al. 2018), as well as methods for testing distribution shifts (Rabanser et al. 2019). We implement a version of the reliability score, the SWIF(r) Reliability Score or SRS, and illustrate the utility of the reliability score using multiple biological datasets. The SRS is able to operate in the presence of missing attribute values, a strength that sets it apart from many related methods[...] Most existing methods for the detection of outliers and novel instances in datasets operate in the second mode, providing a secondary analysis that creates a new model of the data, and then filters the data based on this model (Rousseeuw and Van Driessen, 1999; Breunig et al. 2000; Liu et al. 2008, Liu et al. 2012, Pimentel et al. 2014). Methods for detecting out-of-distribution instances in the context of neural networks (Liang et al. 2017; DeVries and Taylor 2018; Lee et al. 2018) bear some resemblance to the SRS, as they attempt to use aspects of the neural network architecture to derive measures of confidence (DeVries and Taylor 2018) or to identify out-of-distribution instances (Liang et al. 2017).”

4. The paper needs to expand the section explaining SWIF(r) and SRS, currently, the explanation is not self-sufficient and the reader needs to go back to the SWIF(r) manuscript to properly understand the contribution of the current work.

We have expanded the text in **Overview and Definition of SRS** to ameliorate this:

“Briefly, from simulated data for each class, SWIF(r) learns a joint, two-dimensional Gaussian mixture for each pair of attributes $f(X_{i}=x_{i}, X_{j}=x_{j} | class)$, along with one-dimensional marginal Gaussian mixtures for each attribute $f(X_{i}=x_{i} | class)$. From the joint distributions, we derive conditional distributions $f(X_{i}=x_{i} | X_{j}=x_{j}, class)$. We then express the likelihood of the class given n attributes as follows:$L(class | {X_{1}=x_{1}},...,{X_{n}=x_{n})}\approx\sum_{j=1}^{n} [f({X_{j}=x_{j}}| class)\prod_{i \neq j} f({X_{i}=x_{i}}| X_{j}=x_{j} , class)]$ (Equation 1) Where $X_{i}$ represents the i^th^ attribute and n is the number of attributes. With the use of prior class probabilities, and a normalization factor representing the marginal probability of the data summed over all classes, SWIF(r) returns a posterior probability for each class (see Materials and Methods, Equation 3).”

Including an updated section in **Materials and Methods** using more standardized notation for consistency with the previous section**:**

SWIF(r) was originally defined to calculate the probability that a genomic site is adaptive (vs neutral) using the following formula, with the terms adjusted here to suit the more general case and to match the definition of the SRS:

$P(class 1|X_{1} = x_{1}, ..., X_{n}= x_{n}) = \frac{\pi\sum_{j=1}^{n} [f({X_{j}=x_{j}}|class 1)\prod_{i \neq j} f(X_{i}=x_{i}| X_{j}=x_{j} ,class 1)]}{[\pi\sum_{j=1}^{n} [f({X_{j}=x_{j}}| class 1)\prod_{i \neq j} f({X_{i}=x_{i}}| X_{j}=x_{j} , class 1)] + (1-\pi)\sum_{j=1}^{n} [f({X_{j}=x_{j}}| class 2)\prod_{i \neq j} f({X_{i}=x_{i}}| X_{j}=x_{j} , class 2)]}$ (Equation 3)

We have also added the following text to the **Introduction** to provide more intuition about SWIF(r) and the SRS:

“SWIF(r) returns posterior class probabilities based on learned joint distributions of attributes (selection statistics in our motivating application) and user-provided priors on the class frequencies. The probabilities output by SWIF(r), and by other probabilistic classifiers, represent a posterior probability distribution across classes, with probabilities summing to 1 regardless of how well a classified instance matches the training data. As a consequence, a high classification probability can be untrustworthy for instances that do not resemble any of the classes included in training, while an outcome assigning equal probability to all classes may nevertheless be trustworthy if the classifier has been trained using many similar instances. When using a typical classifier, users will receive classification outcomes from their models, with little visibility into the underlying features contributing to classification [(Murphy 2012; Rudin et al. 2022)](https://paperpile.com/c/mCWVvz/5HQ8+m0vv) and thus the trustworthiness of individual classification decisions; the purpose of the SRS is to provide this important feedback.”

Minor comments:

1. The references to figure 1 in the text are incorrect. In the section, Overview and Definition of SRS, the text refers to:

a. Figure 1B instead of Figure 1A.

We thank the reviewer for catching this, and we have fixed it.

b. The two classes are labeled as class 1 and class 2 in Figure 1A but referred to as classes A and B in the text.

We thank the reviewer for catching this, and we now refer to the classes as “class 1” and “class 2” throughout.

c. The second panel of Figure 1B shows SWIF(r) probability and the third panel shows the SRS score however, the text has the two references flipped.

We thank the reviewer for catching this, and we have fixed it.

2. Figure 2 panels are not probably explained in the text.

We have added text to **The SRS is sensitive to instances from unknown classes** that now includes specific references to relevant panels to aid in interpretation:

“In order to demonstrate the effect of missing classes in the training set in an authentic biological context, we analyzed a dataset containing seven morphological attributes of seeds from three types of wheat (Figure 2A, S1). We investigate the performance of SWIF(r) and the SRS in the case where one of the three classes is unrepresented in training, but present in testing. Figure 2B-C show that for each model, the instances belonging to the class that was excluded from training have significantly lower SRS values than instances that belong to a known class (p-value < 1e-08, Mann-Whitney Test; Table S1). This is even true when the unknown class contains values that are intermediate to the two known classes (Figure 2B, leftmost panel), suggesting that the SRS is able to indicate deficiencies in the training set without relying on the presence of extreme attribute values. When SWIF(r) is trained on all three classes, no class has significantly different SRS values from the other classes (p-value > 0.05, Mann-Whitney Test; Table S1, Figure S2). We note the roughly bimodal shape of the SRS scores when a class missing from training data is represented by many instances in testing data. Combining the bimodal profile with domain expertise to inspect the low-SRS samples and identify their common features could lead to the identification of a novel class.”

Reviewer #2: - This work presents a novel, generalizable metric “reliability score” (or “SRS” in this particular application), used to diagnose several types of issues in machine learning workflows, especially those due to differing contexts between training and testing data (missing classes, cohort effects, and missing attributes). Its successful application to three distinct datasets promises broad utility in supporting ML analyses. However, some claims regarding the SRS require additional control experiments to determine whether differences in SRS distributions are due to limitations in the training data or genuine class/cohort differences.

- SRS definition, Paragraph 4: In Model Y in Figure 1B, the area directly between the centers of the two classes cannot be classified (SWIF(r) probability ~ 0.5), yet the SRS for that area is relatively high. The results text focuses on cases with high SWIF(r) probability but low SRS, and requires an interpretation of this reversed example with low SWIF(r) probability but high SRS for a complete picture on how to interpret the possible combinations of prediction probabilities and SRSs.

We thank the reviewer for this comment, and are happy to have the opportunity to explain this more clearly. We have added text to **Overview and Definition of the SRS** that walks through two scenarios where SWIF(r) probability is around 0.5: where the SRS is high, and where the SRS is low to illustrate this point. The full excerpt is below:

“Since SWIF(r) tries to find the “best” class to fit a particular instance, we see instances that lie far from the training data of both classes, as captured by the SRS, that are nevertheless classified with high probability to one class or the other. More generally, this illustrates the inherent limitations of a classifier as a set of decision boundaries. On the other hand, we can consider instances with SWIF(r) probabilities close to 0.5: in some cases, the SRS is low for these instances (Figure 1, panel B, left), while in other cases the SRS is high (Figure 1, panel B, center). For instances where SWIF(r) probability is close to 0.5 and the SRS is low, we have clearly separated distributions for each class, and points with SWIF(r) probability close to 0.5 are merely intermediate between the two classes (Figure 1, panel B, left) while being a poor fit to both. When SWIF(r) probability is close to 0.5 and SRS is high, this indicates that there is overlap in the distributions of the training data within which instances are well-fit by both classes, and are therefore ambiguous and difficult to classify (Figure 2, panel B, center). Combining SWIF(r) classification output with the SRS enables a more complete and nuanced interpretation of results.”

- SRS and unknown classes, Paragraph 1: It’s not clear why the specific attribute pairs in Figure 2A and 2C were selected to represent the wheat data. If the aim is to show broadly how the data are distributed, alternate approaches include projecting the data onto the first two components from PCA to capture maximum variance, or generating all individual and pairwise attribute plots (i.e. Seaborn “pairplot”) as a supplementary figure. Please address or clarify.

We have added a new display item -- **Figure S1** -- that contains a pairplot with all 2-dimensional views into the dataset. We have retained the particular plots in Figure 2 as examples, since they aid in interpretation of the SWIF(r) and SRS values.

- SRS and unknown classes, Paragraph 1: This section and Figure 2B-C suggests a strong separation in SRS between predictions for instances from classes known vs unknown to the training data. Please include statistical tests (i.e. Mann-Whitney) to quantify the significance of this separation in SRS values between known vs. unknown classes and known vs. known classes. Additionally, please include similar SRS plots for a SWIF(r) model trained with all three classes labeled as a control model and discuss whether the SRS values per class become less separable with full label information or if the difference persists (possibly due to genuine class-effects on SRS values).

We thank the reviewer for this suggestion. We have included the results of Mann-Whitney tests to quantify the differences between known/unknown and known/known classes. The full results are presented in new display item **Table S1**, and show that there are significant differences in SRS distributions when one of the classes is the missing class. If both classes are known, *or if the model is trained with all 3 classes*, the distribution differences are no longer significant. A histogram of SRS scores when SWIF(r) is trained on all three classes is provided in new display item **Figure S2.** The following excerpt is from **The SRS is sensitive to instances from unknown classes:**

“Figure 2B-C show that for each model, the instances belonging to the class that was excluded from training have significantly lower SRS values than instances that belong to a known class (p-value < 1e-08, Mann-Whitney Test; Table S1). This is even true when the unknown class contains values that are intermediate to the two known classes (Figure 2B, leftmost panel), suggesting that the SRS is able to indicate deficiencies in the training set without relying on the presence of extreme attribute values. When SWIF(r) is trained on all three classes, no class has significantly different SRS values from the other classes (p-value > 0.05, Mann-Whitney Test; Table S1, Figure S2).”

- SRS and systemic bias, Paragraph 1: Similar to Figure 2A, include a figure showing systemic differences between the cohorts (Male vs Female, European vs African) in attribute space preceding Figure 3A (for example, see previous comment on PCA projections).

We have added two new display items to address this: **Figures** **S6** (sex) and **S7** (ancestry). Because of the large number of attributes, we show the data projected onto the first 10 PCs. We have added text to the Figure 3 caption that points to these figures:

“SRS and SWIF(r) probability are calculated over a plane defined by two of the twenty-two model attributes (Hemoglobin v Cholesterol for the sex-mismatch analysis, and BMI v LDL for the ancestry-mismatch analysis), providing a background of points for each graph. For other views into the data, see S6 Fig and S7 Fig.”

- SRS and systemic bias, Paragraph 1: This section and Figure 3A-B suggests that SRS distributions may be able to diagnose systemic bias when applying a model to data from a different cohort than the training set. Similar to before, please add corresponding figures for control models trained with data from both cohorts and discuss whether the mixed training data yields smaller differences in SRS values and/or misclassification rates between cohorts.

We have added four display items to address this: **Figures S8 and S9** contain histograms and boxplots of the SRS scores when SWIF(r) is trained on both sexes (**S8**) and both ancestries (**S9**), with statistics that show less separation between the SRS distributions than in the mis-specified training case. **Figures S10 and S11** contain confusion matrices that show the misclassification rates for classifiers trained on these mixed training datasets. The following excerpt comes from **“The SRS is sensitive to systemic bias in training data relative to application data**:

“When SWIF(r) is trained with data from both the matching and non-matching cohorts, the differences in the SRS are no longer significant for the experiment looking at sex (Figure S8). The difference between the SRS for African and European cohorts is still significant for individuals in the Normal class, though not for individuals in the Elevated class (Figure S9). We do not necessarily see an improvement in overall performance of the classifier with an increase in the diversity of the samples (Figure S10, S11). In fact, the classifier trained on both African and European ancestries performed worse on the European cohort, and only slightly better on the African cohort than the model trained on just the European cohort. This suggests that meaningful differences between the cohorts may be driving misclassification, for example a shift in which phenotypic measurements provide the best indicators of elevated HBA1C in a given population.”

- SRS and missing attributes, Paragraph 3: This section and Figure 4B suggests that the SRS can distinguish informative from uninformative attributes. I would be interested in seeing a more fine-grained assessment of this capability in addition to the informative vs. pure noise test provided. For example, this could involve selecting all instances with 3/5 attributes missing, separating them into 10 groups based on which exact set of 3 attributes are missing, and then comparing the SRS distributions between those 10 groups, to test if SRS can distinguish how informative different subsets of attributes are beyond a simple missingness filter.

We appreciate this idea for analysis, but unfortunately we are fundamentally unable to implement this because of the structure of the data. The missingness in this application is highly non-random, in particular with respect to which attributes are missing. Some attributes are defined all of the time, and others are missing nearly half the time (typically at the same time as others, and for similar reasons, including low minor allele frequency, and proximity to the end of a sequenced region). Therefore, there are combinations of attributes that will never be missing simultaneously, while some will always be missing together.

Minor comments:

*SRS definition, Paragraph 3: Classes are referred to as “class A” and “class B”, while Figure 1/Table S2 refers to them as “class 1” and “class 2”.

We thank the reviewer for catching this, and it has been fixed.

*SRS and unknown classes, Paragraph 1: Expand introduction of wheat dataset and include dataset size (number of instances per wheat class, in training/testing datasets)

We have added **Table S4**, which contains the training and testing dataset sizes for each class. In addition, we have added text to **The SRS is sensitive to instances from unknown classes**:

“In the original dataset from Charytanowicz *et al.* (2010), three varieties of wheat -- Kama (wheat 1), Rosa (wheat 2) and Canadian (wheat 3) -- were grown and visualized using a soft X-ray technique, measuring area, perimeter, compactness, length of kernel, width of kernel, asymmetry coefficient and length of kernel groove. We divided the dataset into training and testing sets, then created three models, each trained on two types of wheat, and tested on all three. We then investigate the performance of SWIF(r) and the SRS in the case where one of the three classes is unrepresented in training, but present in testing.”

*SRS and systemic bias, Paragraph 1: Include dataset size (number of Elevated and Normal instances for Male/Female and European/African cohorts), possibly as table that could be referenced both here and in the Methods.

We have added a display item, **Table S5**, with the dataset sizes for each cohort, which we reference in the relevant Methods section.

*Methods, SRS, Paragraph 1: Equation 3 could be easier to understand if “adaptive”/”neutral” were replaced with “class 1”/”class 2” and the “S” terms replaced with “X” terms, to be more consistent with Equations 1/2 and Figure 1.

We have implemented this change, with preceding text to explain the differences with the formulation in the SWIF(r) manuscript:

“SWIF(r) was originally defined to calculate the probability that a genomic site is adaptive (vs neutral) using the following formula, with the terms adjusted here to suit the more general case and to match the definition of the SRS:

$P(class 1|X_{1} = x_{1}, ..., X_{n}= x_{n}) = \frac{\pi\sum_{j=1}^{n} [f({X_{j}=x_{j}}|class 1)\prod_{i \neq j} f(X_{i}=x_{i}| X_{j}=x_{j} ,class 1)]}{[\pi\sum_{j=1}^{n} [f({X_{j}=x_{j}}| class 1)\prod_{i \neq j} f({X_{i}=x_{i}}| X_{j}=x_{j} , class 1)] + (1-\pi)\sum_{j=1}^{n} [f({X_{j}=x_{j}}| class 2)\prod_{i \neq j} f({X_{i}=x_{i}}| X_{j}=x_{j} , class 2)]}$ (Equation 3)”

*Methods, SRS, Paragraph 2: Classes are referred to as “class A” and “class B”, while Figure 1/Table S2 refers to them as “class 1” and “class 2”.

We thank the reviewer for catching this, and it has been fixed.

*Figure 1B: Add axis labels, “attribute 1” and “attribute 2”

This figure has been updated to add these labels.

*Figure 2C: Add axis labels, at least to the True Labels plot.

This figure had been updated to add these labels.

*Figure 3A: X-axes four all four panels seem zoomed out, zooming in on peaks could better show the separation in SRS values.

We note that we have retained the histograms as-is because we believe the large range of SRS values is relevant, but we also note that the boxplots are provided precisely to show this separation more clearly. We have added text to the figure caption that makes this point clearer:

Histograms and boxplots showing the distributions of “match” and “mismatch” cohorts for each experiment. Histograms and boxplots show the same information, with boxplots zoomed in to allow for easier comparison of the distributions. The x-axis range in the histograms represents the complete range of observed SRS values in each case.

*Figure 3C: Add axis labels.

These axis labels appear directly above the plots for ease of visualization, and we have added text to the Figure 3 caption that emphasizes this, in addition to pointing to the supplementary figures containing the PCA projections:

“SRS and SWIF(r) probability are calculated over a plane defined by two of the twenty-two model attributes (Hemoglobin v Cholesterol for the sex-mismatch analysis, and BMI v LDL for the ancestry-mismatch analysis), providing a background of points for each graph. For other views into the data, see S6 Fig and S7 Fig.”

*Figures 1-3 need larger axis tick labels.

Since axis values in these figures often refer to standardized values, or SRS values which are not meant to be compared cross-experiments, we are opting to de-emphasize these tick labels to keep the focus of these figures on relative values, and retain space for important legends and labels. All tick marks could be eliminated without loss of information, but we retain them so that they are available for inspection in digital formats.

Reviewer #3: In this work, the authors come up with a per-exemplar score called SRS by fitting a generative classifier (specifically an Averaged One-Dependence Estimation model) to labeled data. The SRS is then defined as the max log likelihood over classes. The authors demonstrate the general applicability of SRS as a tool to detect outliers and differences in training and application data.

By itself, the work is sound and the applications shown are reasonable and useful. However, my primary concern with this work is that it fails to discuss and/or compare against relevant literature in this vast field. The claim that SRS is a "new concept for scientific machine learning studies" lacks support. A particularly similar idea to SRS is using likelihood from mixture models to detect outliers (see <https://scikit-lego.readthedocs.io/en/latest/mixture-methods.html#outlier-detection>). SRS is essentially a simpler version of this where the class labels are known (uses max likelihood over classes instead of likelihood).

We appreciate the reviewer pointing us in the direction of relevant literature, and we have worked to place our manuscript in context and perform appropriate comparisons with existing methods. We have also softened the language around novelty to make it clear how the SRS fits into the existing landscape.

Briefly, we posit that the SRS is valuable in the quest for machine-learning transparency, and exists in the spirit of other techniques for introducing abstention into models. It differs from other methods of abstention in that it does not require the addition of an abstention class or manipulation of a loss function; instead it can rely directly on the data model underlying the classifier, producing an interpretable measure of “trustworthiness”. The SRS shares some similarities with methods for outlier/novelty detection, and we show that it generally has similar performance at detecting unexpected instances, although the SRS has the added benefit of being able to operate in the presence of missing data (instances for which not all attributes are defined). In addition, we explore how the SRS relates to tools for detecting distribution shift; we argue that the SRS can be useful as a dimensionality-reduction tool in looking for differences between training and testing data; the determination of significance can then be left to two-sample tests of various types. We also posit that there is utility in the SRS being packaged as a default output with every classified instance, without the need for additional post-hoc analysis.

Please refer here to our response to point #3 from Reviewer #1, which includes references to the new display items (**Figures S2, S3, and S4**) in which we compare the SRS to three methods for novelty detection, as well as excerpts from the **Introduction, Results,** and **Discussion** where discuss the relationship between the SRS and outlier/novelty detection methods, methods for detection of distribution shift, and the literature about abstention in machine learning classifiers.

Finally, We note that we cannot access the link provided above, but we think the comparisons we have performed (including some suggested in the reviewer’s next comment) are useful for putting our method in context.

The work has close ties to multiple areas, none of which are discussed in the paper:

- outlier/novelty detection: Many methods available here <https://scikit-learn.org/stable/modules/outlier_detection.html>. Another related idea is one class SVM: <https://www.microsoft.com/en-us/research/wp-content/uploads/2016/02/tr-99-87.pdf>. Longer review <https://www.robots.ox.ac.uk/~davidc/pubs/NDreview2014.pdf>.

- testing differences between high dimensional distributions (aka two-sample test): See <https://normaldeviate.wordpress.com/2012/07/14/modern-two-sample-tests/> for a helpful overview of methods such as MMD (<https://www.jmlr.org/papers/volume13/gretton12a/gretton12a.pdf>), cross-match test.

- abstention/robustness/out-of-distribution and domain shift/adaptation in ML: see e.g. <https://arxiv.org/abs/2105.07107>, <https://arxiv.org/abs/1802.04865>, <https://arxiv.org/abs/1706.02690>, <https://arxiv.org/abs/1807.03888>

We hope that the additional analysis, figures, and text described above serve to address these comments. In particular, we have added analyses comparing the SRS to multiple off-the-shelf methods for novelty/outlier detection as described above, and we place the SRS in context with respect to the question of detecting distribution shift between training and testing data, with references to many of these papers provided by the reviewer. In addition, we demonstrate how the SRS can be used as a dimensionality reduction step prior to the use of two-sample tests to look for significant shifts: see for instance, the p-values now provided along with **Figure 2** and **Figure 3** and in new display items **Table S1**, and **Figures S8 and S9**.

Overall, it is not clear what the primary contribution of the proposed method is over the plethora of alternatives that serve a similar function. It would be helpful to contextualize this work and discuss the pros and cons over existing methods.

We thank the reviewer again for these suggestions; we think the manuscript is genuinely stronger for each piece of added contextualization and comparison described above.

Reviewer #4: The authors discussed a metric, reliability score (SRS), to assess the reliability of classification of a given instance. Specifically, the authors point out three importance challenge in machine learning that SRS can help resolve: 1) an unknown class present in testing data that was not present in training data, 2) systemic mismatch between training and testing data, and 3) instances of testing data that are missing values for some attributes. I have three major comments that I hope that authors can provide more explanations on.

First, there is no question that, when we know the ground truth in either cases, SRS can help distinguish the presence of an unknown class, or indicate the presence of systemic bias. However, one of the most challenging issues is that frequently we cannot distinguish 1) and 2). When we have poorly classified test instances, we almost always do not know if it is a new class or the training data simply cannot lead to a more general model. My understanding is that SRS cannot help us in this regard. So, the claim that SRS can help resolve 1) and 2) only applies when we know a prior what the situation is, which make it not particularly useful.

We have worked to address these comments in a few ways. First, we think there are particular instances in which the distribution of the SRS itself can provide some clues. In particular, the following is an excerpt from **The SRS is sensitive to instances from unknown classes:**

“We note the roughly bimodal shape of the SRS scores when a class missing from training data is represented by many instances in testing data. Combining the bimodal profile with domain expertise to inspect the low-SRS samples and identify their common features could lead to the identification of a novel class.”

In addition, the question of systemic bias is often one that can be plausibly predicted; for example, in moving from simulated testing data to real-world data, or in trying to use a trained classifier on a new testing set from a new population or source. In this case, the distribution of SRS values can serve to assess the generalizability of the trained classifier. The following is an excerpt from **The SRS is sensitive to systemic bias in training data relative to application data:**

“This experiment represents an important use case for the SRS; in many contexts, researchers may be unsure how well their classifiers will generalize to a new dataset. The SRS provides a quick way to view the overall difference in model fit between a training or validation set and a testing set, making it straightforward to test for significant shifts in SRS distribution using two-sample tests (Rabanser et al. 2019), and to subsequently make decisions about the need for additional training.”

In situations that do not align with one of the above cases, we agree that this is a fundamentally difficult problem, and one that really relies on domain-specific knowledge and thoughtful use of the SRS. We have added text to the **Discussion** that explores the nuances of this issue:

“One limitation of the SRS is that while the SRS indicates problems with missing data or deficiencies in the dataset, a solution to those problems may not always be possible or simple to implement. Researchers will need to rely on domain-specific knowledge to decide whether the indicated problem can be solved by removing instances, adding additional classes or attributes to their model, or by including more, or more diverse instances, in their training data. It may also be advantageous to train independent classifiers for cohorts that may have distinct underlying properties, for example, training separate classifiers for each sex in a study where the goal is to distinguish individuals with a disease phenotype (Mostafavi et al. 2020, Figure 3). Discovering a solution to low-reliability instances will require answers to subjective and context-dependent questions that may be difficult to anticipate and may not generalize. Care should be taken to use abstention, or the removal of instances in a principled and appropriate manner. When there is risk involved in making the wrong classification, abstention can be an important practice, as in the medical context (Herbei and Wegkamp 2009; Gandouz et al. 2021). In other cases there may be a distinctive factor impacting the performance of the classifier on specific instances: for example, when simulated training data fails to capture dynamics in unusual genomic regions. Abstention or removal of instances should not be used to boost measurement of performance of the classifier, but to invite further investigation and scrutiny. When distribution shifts or additional classes are suspected, researchers may face decisions about the number and types of classes to include in training, and cost-benefit considerations when deciding whether to gather the additional data required to add new attributes or additional training examples. Increasing transparency and publication of models with negative results would help address some of the problems raised by these subjective questions [...] Domain-specific biological knowledge currently provides the best available guide for navigating the complex, multi-factorial choices involved in model design. In order to best leverage this domain-specific expertise, we need to ensure that machine learning models are easy to interpret, dissect and critique, even by researchers without machine learning expertise.”

Second, the SRS appears useful in identifying instances that do not resemble training data. However there are many other measures one can use, including very simple ones such as Euclidean distance or similarity measures like correlation, to achieve the same goal. SRS’ calculation requires priors and calculation can be involved. Is it better than the other approaches? If so, can there be some ways to formally demonstrate this?

Thanks to this comment, and similar comments from the other reviewers, we now include comparisons to other methods that perform outlier or novelty detection. Please refer here to our response to point #3 from Reviewer #1, which includes references to the new display items (**Figures S2, S3, and S4**) in which we compare the SRS to three methods for novelty detection, as well as excerpts from the **Introduction, Results,** and **Discussion** where discuss the relationship between the SRS and outlier/novelty detection methods, methods for detection of distribution shift, and the literature about abstention in machine learning classifiers.

Third, I would love to have some reliability measure for each instance in my classification tasks. But given the first point, while I appreciate the conceptual basis of a reliability score, SRS seems to be an outlier detection measure where there are already plenty.

We hope our answers above support the utility of the SRS. In particular, we point to a couple of excerpts from the **Introduction** and **Discussion** where we make this argument:

“By packaging our classifier and the SRS together, we prioritize and highlight the need for evaluations of outliers and distribution differences to be included in every machine learning analysis. Rather than performing outlier detection or looking for distribution differences prior to performing classification, or as a post-hoc analysis, researchers get SRS output and SWIF(r) classification output in a single step. Exclusion of outliers and many out-of-distribution instances can be performed by setting a threshold on the SRS, in much the same way that other methods for outlier and novelty detection can be applied. The SRS can also be used to measure distribution shifts, fitting into the “Failing Loudly” framework for machine learning described in Rabanser et al. 2019, in which the authors advocate for machine learning tools that give warnings when facing unexpected input. In this particular framework, the SRS can serve the role of dimensionality reduction, a step in the Failing Loudly pipeline prior to testing for significant distribution shift with subsequent two-sample tests.”

“The reliability score shares some features with outlier detection, novelty detection and detecting out-of-distribution instances (Rousseeuw and Van Driessen, 1999; Breunig et al. 2000; Liu et al. 2008, Liu et al. 2012, Pimentel et al. 2014, DeVries and Taylor 2018, Lee et al. 2018), as well as methods for testing distribution shifts (Rabanser et al. 2019) [...] The SRS is able to operate in the presence of missing attribute values, a strength that sets it apart from many related methods [...] Across many areas of biology there is an opportunity to improve machine learning analyses by creating community standards and best practices for simulation, interpretability and reproducibility. By packaging SWIF(r) and the SRS together into a single workflow, we aim to give researchers the tools to both apply the machine learning method, and to critique and interpret the results they obtain.”

More details:

1. p.2, “instances of testing data that are missing values for some attributes” – should it be “have missing values”?

We have edited this sentence as suggested:

“instances of testing data that have missing values for some attributes.”

2. p.5, “When these differences arise, it may be preferable to set aside data instances with poor fit,

allowing the classifier to effectively abstain when it encounters signatures it has not been trained

to expect, one goal of the SRS is to enable the user to identify such poor-fit instances”: This aspect needs to be more clearly explained and nuances listed. The practice can also make a model looks better than it actually is. It is not straightforward to distinguish true outliers from training data that do not sufficiently represent the true distribution.

We appreciate this point, and we have added the following text to the **Discussion** to emphasize that the goal of identifying poor-fit instances is to give a researcher a better handle on their data, and to point to instances that would benefit from a closer look. In particular, we point out that abstention should not be used to artificially inflate a model’s performance:

“Care should be taken to use abstention, or the removal of instances in a principled and appropriate manner. When there is risk involved in making the wrong classification, abstention can be an important practice, as in the medical context (Herbei and Wegkamp 2009; Gandouz et al. 2021). In other cases there may be a distinctive factor impacting the performance of the classifier on specific instances: for example, when simulated training data fails to capture dynamics in unusual genomic regions. Abstention or removal of instances should not be used to boost measurement of performance of the classifier, but to invite further investigation and scrutiny.”

3. p.7, equation 1: I am confused as to whether the sum term and the multiplication terms are done separately, or P(xi|class) is multiplied with all non-i attributes’ prior individuals, then summed together.

We thank the reviewer for noting the need for clarification. We have added an extra set of parentheses to make this unambiguous. The sum is over all terms $f({X_{j}=x_{j}}| class)\prod_{i \neq j} f({X_{i}=x_{i}}| X_{j}=x_{j} , class)$. This is an approximation of the joint distribution of all attributes, and the sum is effectively an average of these approximations.

4. p.8, “Because the posterior probabilities are forced to sum to 1, instances that do not resemble any trained class may still receive a high classification probability for the “least bad” option…”: The authors then indicated that “SRS is designed to help detect such instances”. In Figure 1B (by the way, 1A would be out of order and has not been introduced yet), the authors nicely shown that some test cases that do not fall within the range of the training data would have poor SRS. So, an instance can have high classification probability but low SRS. The confusing thing here is then what is the “classification probability” the authors speak of? Some clarification here will be helpful to confused readers like myself.

We have resolved the figure panel references. In addition, we have added text to clarify this, and we thank the reviewer for giving us the opportunity to better explain what is an important point. Briefly, the classification probability is the classic output of a (probabilistic) classifier, representing a posterior probability of each class given the data for a particular instance. Since these probabilities sum to 1 over all classes, these probabilities cannot convey whether the class with the highest posterior probability is actually a good fit, or whether it represents the “least bad” option. We have added the text in the **Introduction** to clarify this:

“The probabilities output by SWIF(r), and by other probabilistic classifiers, represent a posterior probability distribution across classes, with probabilities summing to 1 regardless of how well a classified instance matches the training data. As a consequence, a high classification probability can be untrustworthy for instances that do not resemble any of the classes included in training, while an outcome assigning equal probability to all classes may nevertheless be trustworthy if the classifier has been trained using many similar instances. When using a typical classifier, users will receive classification outcomes from their models, with little visibility into the underlying features contributing to classification [(Murphy 2012; Rudin et al. 2022)](https://paperpile.com/c/mCWVvz/5HQ8+m0vv) and thus the trustworthiness of individual classification decisions; the purpose of the SRS is to provide this important feedback.”

Additionally, in **Overview and Definition of the SRS**, we have included the following text to walk through the relationship between classification probabilities from SWIF(r) and the SRS scores:

“Since SWIF(r) tries to find the “best” class to fit a particular instance, we see instances that lie far from the training data of both classes, as captured by the SRS, that are nevertheless classified with high probability to one class or the other. More generally, this illustrates the inherent limitations of a classifier as a set of decision boundaries. On the other hand, we can consider instances with SWIF(r) probabilities close to 0.5: in some cases, the SRS is low for these instances (Figure 1, panel B, left), while in other cases the SRS is high (Figure 1, panel B, center). For instances where SWIF(r) probability is close to 0.5 and the SRS is low, we have clearly separated distributions for each class, and points with SWIF(r) probability close to 0.5 are merely intermediate between the two classes (Figure 1, panel B, left) while being a poor fit to both. When SWIF(r) probability is close to 0.5 and SRS is high, this indicates that there is overlap in the distributions of the training data within which instances are well-fit by both classes, and are therefore ambiguous and difficult to classify (Figure 2, panel B, center). Combining SWIF(r) classification output with the SRS enables a more complete and nuanced interpretation of results.”

5. p.8, another thought about Figure 1B: SRS is a measure that is useful for picking out which instances are similar to training data. That is fine, but we can imagine this can also be done using some simple similarity measures to training instances without the need to calculate posterior probabilities. If that is the case, what is special about SRS?

We point to our explanation above following the broad comments from this reviewer. In particular, we argue that the SRS has a few inherent benefits: First, it is able to be calculated in the presence of instances with missing attributes, which other outlier/novelty methods cannot do. Second, it provides a dimensionality reduction from which tests for distribution shift can be easily calculated, allowing researchers to test the generalizability of their model in the presence of data from a new source. Third, it is packaged as a default option with SWIF(r), with the intention of making this kind of analysis more commonplace in biological machine learning. Putting these three features together, we think that the SRS is an effective way of providing researchers with immediate feedback about their models, pointing to possible remedies and flagging potentially worrisome data points or trends.

6. p.9 and on, the authors discussed the utility of SRS in picking out instances from unknown classes. There is no question that the SRS scores for the unknown classes are lower. But one distinction I’d like to make is that this does not mean that all instances with low SRS scores belong to some unknown classes (this seems to be what the authors are suggesting). Another possibility of low SRS scores can also be attributed to non-representative training data, which would make some test cases look like outliers when they actually are not. The challenge is to distinguish between these two possibilities. Can the authors provide some clarification as to how SRS can be helpful here?

7. p.10, the author indicated that, when there are systematic differences between training and application data, SRS can indicate them. Again, there is no question that SRS can show that there are systematic differences as Figure 3 shows when we know that is the case. The issue is however, when we do not know, how can one use SRS to distinguish the possibility between unknown class (point 6) and systematic bias.

In response to points 6 and 7, we refer to our response to the broader comments from this reviewer. We completely agree that low SRS scores can reflect a variety of causes, including unknown classes, and non-representative or biased training data. The text we added and referenced above points to a few possible use cases and patterns: in particular, the bimodal pattern associated with a missing class containing a large number of attributes in the test data, and the use case in which a researcher uses a test of SRS distribution shift to assess the generalizability of a classifier to new data (including whether additional or different training data is required). In addition, outlier SRS values encourage the researcher to take a closer look at particular instances and potentially catch artifacts that would otherwise go unnoticed. We also reiterate that this is a difficult, but important problem in general, and point again to the added text from the Discussion that we reference above, in which we emphasize the importance of domain-specific knowledge.

**Have the authors made all data and (if applicable) computational code underlying the findings in their manuscript fully available?**

The [PLOS Data policy](https://journals.plos.org/ploscompbiol/s/materials-and-software-sharing) requires authors to make all data and code underlying the findings described in their manuscript fully available without restriction, with rare exception (please refer to the Data Availability Statement in the manuscript PDF file). The data and code should be provided as part of the manuscript or its supporting information, or deposited to a public repository. For example, in addition to summary statistics, the data points behind means, medians and variance measures should be available. If there are restrictions on publicly sharing data or code —e.g. participant privacy or use of data from a third party—those must be specified.

Reviewer #1: **No:** The code is not available at the provided link (<https://github.com/ramachandran-lab/SRS_paper>)

Reviewer #2: Yes

Reviewer #3: Yes

Reviewer #4: Yes

PLOS authors have the option to publish the peer review history of their article ([what does this mean?](https://journals.plos.org/ploscompbiol/s/editorial-and-peer-review-process#loc-peer-review-history)). If published, this will include your full peer review and any attached files.

If you choose “no”, your identity will remain anonymous but your review may still be made public.

**Do you want your identity to be public for this peer review?** For information about this choice, including consent withdrawal, please see our [Privacy Policy](https://www.plos.org/privacy-policy).

Reviewer #1: No

Reviewer #2: No

Reviewer #3: No

Reviewer #4: No

Figure Files:

While revising your submission, please upload your figure files to the Preflight Analysis and Conversion Engine (PACE) digital diagnostic tool, [https://pacev2.apexcovantage.com](https://pacev2.apexcovantage.com/). PACE helps ensure that figures meet PLOS requirements. To use PACE, you must first register as a user. Then, login and navigate to the UPLOAD tab, where you will find detailed instructions on how to use the tool. If you encounter any issues or have any questions when using PACE, please email us at figures@plos.org.

Data Requirements:

Please note that, as a condition of publication, PLOS' data policy requires that you make available all data used to draw the conclusions outlined in your manuscript. Data must be deposited in an appropriate repository, included within the body of the manuscript, or uploaded as supporting information. This includes all numerical values that were used to generate graphs, histograms etc.. For an example in PLOS Biology see here: <http://www.plosbiology.org/article/info%3Adoi%2F10.1371%2Fjournal.pbio.1001908#s5>.

Reproducibility:

To enhance the reproducibility of your results, we recommend that you deposit your laboratory protocols in [protocols.io](http://protocols.io/), where a protocol can be assigned its own identifier (DOI) such that it can be cited independently in the future. Additionally, PLOS ONE offers an option to publish peer-reviewed clinical study protocols. Read more information on sharing protocols at <https://plos.org/protocols?utm_medium=editorial-email&utm_source=authorletters&utm_campaign=protocols>
